# Expression of long non‐coding RNA ENSG00000226738 (LncKLHDC7B) is enriched in the immunomodulatory triple‐negative breast cancer subtype and its alteration promotes cell migration, invasion, and resistance to cell death

**DOI:** 10.1002/1878-0261.12446

**Published:** 2019-02-09

**Authors:** Fredy Omar Beltrán‐Anaya, Sandra Romero‐Córdoba, Rosa Rebollar‐Vega, Oscar Arrieta, Verónica Bautista‐Piña, Carlos Dominguez‐Reyes, Felipe Villegas‐Carlos, Alberto Tenorio‐Torres, Luis Alfaro‐Riuz, Silvia Jiménez‐Morales, Alberto Cedro‐Tanda, Magdalena Ríos‐Romero, Juan Pablo Reyes‐Grajeda, Elda Tagliabue, Marilena V. Iorio, Alfredo Hidalgo‐Miranda

**Affiliations:** ^1^ Laboratorio de Genómica del Cáncer Instituto Nacional de Medicina Genómica Mexico City Mexico; ^2^ Programa de Doctorado en Ciencias Biomédicas Facultad de Medicina Universidad Nacional Autónoma de México (UNAM) Mexico City Mexico; ^3^ Department of Experimental Oncology and Molecular Medicine Istituto Nazionale dei Tumori Milan Italy; ^4^ Thoracic Oncology Unit Instituto Nacional de Cancerología (INCan) Mexico City Mexico; ^5^ Instituto de Enfermedades de la Mama FUCAM Mexico City Mexico; ^6^ Unidad de Proteómica Médica Instituto Nacional de Medicina Genómica Mexico City Mexico

**Keywords:** *ENSG00000226738*, invasion, LncKLHDC7B, long non‐coding RNA, migration, triple‐negative breast cancer

## Abstract

Triple negative breast cancer (TNBC) represents an aggressive phenotype with poor prognosis compared with ER, PR, and HER2‐positive tumors. TNBC is a heterogeneous disease, and gene expression analysis has identified seven molecular subtypes. Accumulating evidence demonstrates that long non‐coding RNA (lncRNA) are involved in regulation of gene expression and cancer biology, contributing to essential cancer cell functions. In this study, we analyzed the expression profile of lncRNA in TNBC subtypes from 156 TNBC samples, and then characterized the functional role of *LncKLHDC7B* (*ENSG00000226738*). A total of 710 lncRNA were found to be differentially expressed between TNBC subtypes, and a subset of these altered lncRNA were independently validated. We discovered that *LncKLHDC7B* (*ENSG00000226738*) acts as a transcriptional modulator of its neighboring coding gene *KLHDC7B* in the immunomodulatory subtype. Furthermore, *LncKLHDC7B* knockdown enhanced migration and invasion, and promoted resistance to cellular death. Our findings confirmed the contribution of *LncKLHDC7B* to induction of apoptosis and inhibition of cell migration and invasion, suggesting that TNBC tumors with enrichment of *LncKLHDC7B* may exhibit distinct regulatory activity, or that this may be a generalized process in breast cancer. Additionally, *in silico* analysis confirmed for the first time that the low expression of *KLHDC7B* and *LncKLHDC7B* is associated with poor prognosis in patients with breast cancer.

**Abbreviations**
ADAM9: ADAM metallopeptidase domain 9
AMOT: angiomotin
APP: amyloid beta precursor protein
BL1: basal‐like 1
BL2: basal‐like 2
CAV1: caveolin 1
CCAR2: cell cycle and apoptosis regulator 2
CDH3: cadherin 3
CI: confidence interval
ECM: extracellular matrix
EPS8: epidermal growth factor receptor pathway substrate 8
ER: estrogen receptor
FDR: false discovery rate
FFPE: formalin‐fixedparaffin‐embedded
GAPDH: glyceraldehyde‐3‐phosphate dehydrogenase
GBA: guilt‐by‐association analysis
GEO: gene expression omnibus
HER2: human epidermal growth factor receptor 2
HR: hazard ratio
HTA: human transcriptome array
IM: immunomodulatory
IPA: ingenuity pathway analysis
KLHDC7B: Kelch domain containing 7B
KLHL39: Kelch‐like protein 39
LAR: luminal androgen receptor
LncKLHDC7: long non‐coding KLHDC7B
lncMAP: LncRNA modulator atlas in pan‐cancer
lncRNA: long non‐coding RNA
MHC: major histocompatibility complex
M: mesenchymal
MMP2: matrix metallopeptidase 2
MSL: mesenchymal stem‐like
PMAIP1: phorbol‐12‐myristate‐13‐acetate‐induced protein 1
PR: progesterone receptor
QBS: 2‐amino‐N‐quinolin‐8‐yl‐benzenesulfonamide
RAD21: RAD21 cohesin complex component
shRNA: short hairpin RNA
TCGA: The Cancer Genome Atlas
TGF: transforming growth factor
TN: triple‐negative
TNBC: triple‐negative breast cancer
TNC: tenascin C
TNF: tumor necrosis factor
U87: small nucleolar RNA U87
UNS: unstable

## 1 Introduction

Triple negative breast cancer (TNBC), defined in the clinic by the negative expression of estrogen receptor (ER), progesterone receptor (PR), and lack of overexpression of human epidermal growth factor receptor 2 (HER2), is a heterogeneous tumor that represents ~ 14–23% of all breast cancers (BC) (Lara‐Medina *et al*., 2011; Martinez *et al*., 2013; Perez‐Rodriguez, 2015). TNBC currently lacks targeted treatment, is more aggressive, has worse global survival in metastatic disease, affects young women, and a higher proportion of African American and Hispanic women (Bauer *et al*., 2007; Dent *et al*., 2007; Foulkes *et al*., 2010; Rastelli *et al*., 2010). With the development and advancement of high throughput technologies, TNBC has been classified into intrinsic subtypes based on coding gene expression [messenger (m)RNA] and ontology analysis into the following groups: two basal‐like (BL1 and BL2), immunomodulatory (IM), mesenchymal (M), mesenchymal stem‐like (MSL), luminal androgen receptor (LAR), and unstable (UNS) subtypes (Lehmann *et al*., 2011). Subtyping of TNBC based on non‐coding RNA (ncRNA) expression and research on its therapeutic implications are still being refined (Lehmann *et al*., 2016).

During the development of cancer, major transcriptional alterations can be induced by ncRNA, leading to changes in global gene expression patterns and genomic instability (Wapinski and Chang, 2011). A large proportion of RNAs with no coding capacity (Consortium, 2012), such as long non‐coding RNA (lncRNA) (Martin and Chang, 2012), have emerged as key players in several biological processes. LncRNA have > 200 nucleotides, transcribed by RNA polymerase II, mainly located within the nucleus but also found in the cytosolic compartment, lack protein‐coding potential, and show lower expression compared with mRNA (Derrien *et al*., 2012). The lncRNA species are also considered to be new protagonists in the development of cancer, with potential roles in oncogenic and tumor suppressor pathways (Gibb *et al*., 2011; Li and Chen, 2013; Wapinski and Chang, 2011).

To analyze the differences in lncRNA expression landscapes in TNBC subtypes, we evaluated lncRNA expression profiles of 156 TNBC samples. TNBC subtypes were first defined by mRNA microarray expression profiling, and differentially expressed lncRNA were identified between subtypes. A significant number of differentially expressed lncRNA were identified between all subtypes, and some of them were validated in an independent cohort of TNBC samples. *LncKLHDC7B*, an lncRNA that was over‐expressed in the immunomodulatory subtype, was selected for further investigation of its functional role in TNBC through modulation of its expression in cell lines. Down‐modulation of *LncKLHDC7B* resulted in down‐regulation of its coding gene (*KLHDC7B*) and in the alteration of the expression of several other genes, as well as in an increase in migration and invasion, in addition to resistance to cell death not only in TNBC cell lines, but also in other breast tumor subtypes, suggesting its potential role in breast oncogenesis.

## 2 Materials and methods

### 2.1 Collection and processing of samples

A total of 156 formalin‐fixed, paraffin‐embedded (FFPE) tissues were collected from the Instituto de Enfermedades de la Mama FUCAM (Mexico) and the Istituto Nazionale dei Tumori (Italy) by retrospective collection from 2007 to 2015. Samples were defined as triple‐negative using the current international histopathological criteria, less than 1% of positive cells for ER and PR, and HER2. Immunohistochemical detection of the three markers was carried out using the following antibodies: ER (clone 1D5, Dako, Denmark, Carpinteria, CA, USA), PR (clone PgR636, Dako, Denmark) and HER2 (K5204, Dako). Cytokeratin 5/6 and Ki67 (clone D5/16B4, Dako, Denmark) were also evaluated to rule out potential false‐negative immunohistochemical reactions. All procedures were performed according to the Declaration of Helsinki, and were reviewed and approved by the Ethics and Research Committees of the National Institute of Genomic Medicine (approval number CEI2016/13).

A pathologist identified regions of the sample with the highest amount of cancer tissue, and tissue cores were obtained with a 2‐mm‐wide needle with a tissue arrayer. Tissue cores were used for RNA extraction using the AllPrep® nucleic acid purification FFPE kit (Qiagen, Hilden, Germany) according to the manufacturer's instructions. Subsequently, RNA was quantified by spectrophotometry (Nanodrop, Thermo Fisher Scientific, Waltham, MA, USA) and stored at −80 °C until further processing.

### 2.2 Microarray processing and data analysis

Transcriptional profiles were analyzed using the Affymetrix Human Transcriptome Array V2.0, following the manufacturer's instructions. Briefly, ~ 200 ng of total RNA was converted into complementary (c)DNA and labeled with the SensationPlus™ FFPE Amplification and WT Labeling® kit (Affymetrix, Santa Clara, CA, USA) and hybridized on the array, which detects both mRNA and lncRNA. Arrays were washed, stained, and scanned using a Genechip Scanner 3000 7G (Affymetrix). The data were analyzed with the Robust Multichip Analysis (RMA) algorithm using Affymetrix default analysis settings and global scaling as normalization method on a Affymetrix Transcriptome Analysis Console (tac) V3.0 [Data accessibility gene expression omnibus (GEO): GSE86948]. The ComBat function was used to remove batch effects using the SVA package. The boxplots, heat maps, and correlation analyses were conducted in r software (http://cran.r-project.org; http://www.bioconductor.org; ggplots cran.r‐project.org) using tools from the Bioconductor project (http://www.bioconductor.org) (Wickham, 2009).

### 2.3 Messenger RNA subtyping of triple‐negative breast cancer samples and lncRNA expression profiles

**Figure 1:**
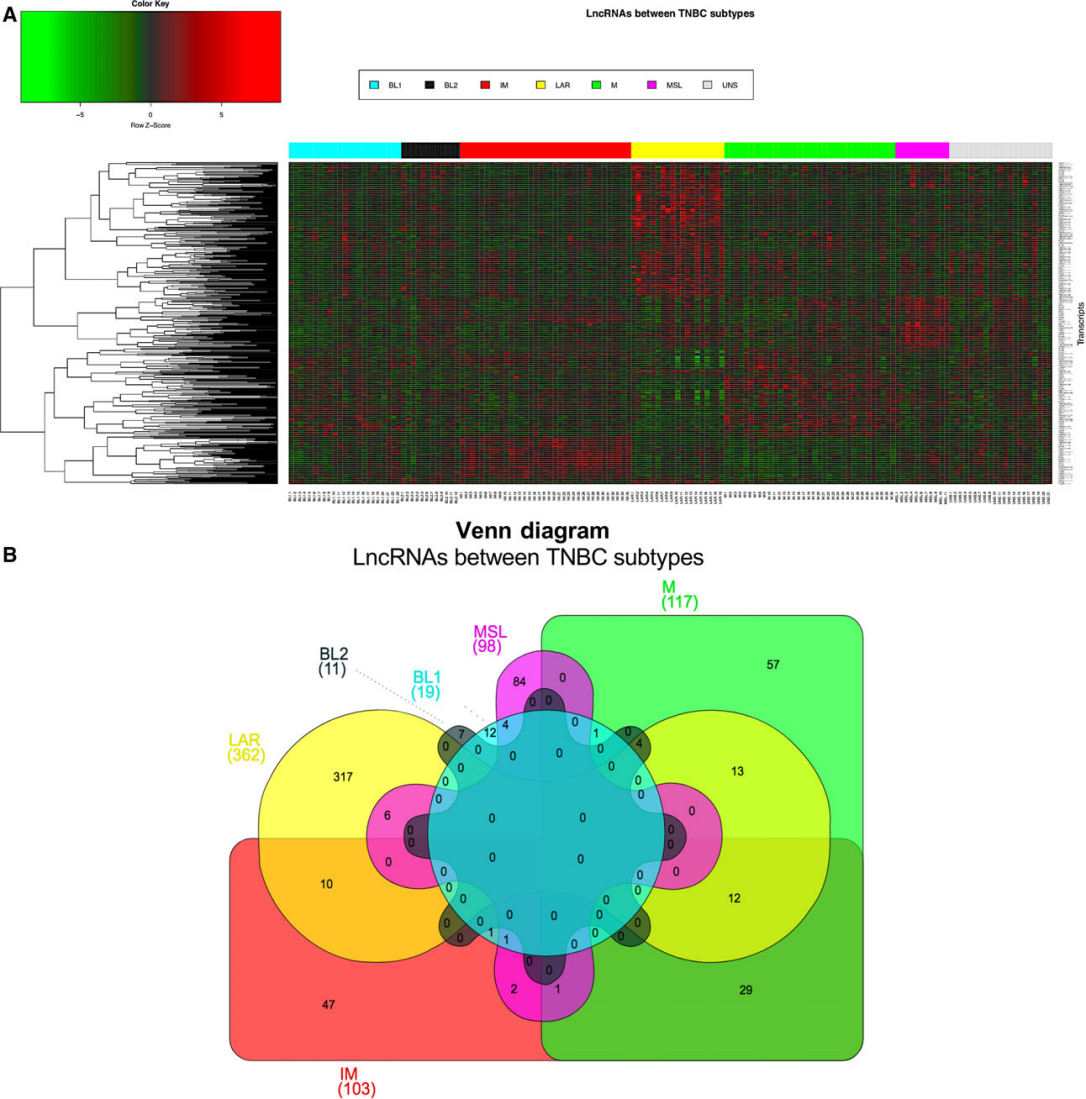
Differential expression of lncRNA across TNBC subtypes. (A) Supervised hierarchical clustering of the differentially expressed lncRNA in TNBC subtypes. Red represents up‐regulation and green down‐regulation. Rows correspond to transcripts and columns to samples, color bar represents each TNBC subtype. (B) Venn diagram shows the common and differential lncRNA between TNBC subtypes.

The Web‐based TNBCtype algorithm (http://cbc.mc.vanderbilt.edu/tnbc/) was used to identify the mRNA‐based subtypes of TNBC in our cases (Chen *et al*., 2012). Four of 160 TNBC samples were excluded from lncRNA expression profile analysis, according to the bimodal filter of the TNBCtype algorithm (Chen *et al*., 2012). After mRNA subtyping, we analyzed the lncRNA expression profiles between subgroups using the Affymetrix Transcriptome Analysis Console (tac) v.3.0, through comparison of the samples belonging to a specific subtype against to the rest of the samples (e.g. IM vs other subtypes). A ≥ 1.5‐fold change, ANOVA *P*‐value less than or equal to 0.05, and false discovery rate (FDR) < 0.05 were considered as significant to detect expression changes between the TNBC subtypes, except for BL2 where FDR was < 0.5. A heatmap with the differentially expressed lncRNA is shown in Fig. 1. TNBC samples from the GEO database (Data accessibility GEO: GSE76250) were used for validation of differential gene expression in an independent cohort (Liu *et al*., 2016). Gene ontology and cellular pathway analysis were carried out using david (Frederick, MD, USA) (Huang da *et al*., 2009). Venn diagrams were used to show the relationship between groups of differentially expressed lncRNA (Heberle *et al*., 2015).

### 2.4 Guilt‐by‐association analysis

Enrichment pathway analysis was carried out by a guilt‐by‐association analysis (Guttman *et al*., 2009). Briefly, normalized gene expression of mRNA and lncRNA of the 156 profiled tumors were used to build a correlation matrix including only the differentially expressed lncRNA and mRNA for each subtype. For each lncRNA, mRNA were ranked according to their Spearman correlation coefficient and *P* value. A significant correlation between lncRNA and mRNA was considered as follows: Spearman correlation > 30% and *P* value < 0.05. The lncRNA‐mRNA co‐expression was computed to define candidate lncRNA prioritization by identifying which transcripts show a coordinated expression pattern across a group of samples. The significant correlated mRNA were then evaluated in an enrichment pathway analysis to define their impact on the signaling process, using enrichr (New York , NY, USA) (Kuleshov *et al*., 2016) to associate the altered lncRNA with pathways and signatures that were significantly enriched. The co‐expression correlations were the basis for constructing an enrichment pathway analysis in which each term represents a set of transcripts with a significant strength co‐expression relationship. To compute this weighted correlation analysis, some functions of the wgcna package were applied (Los Angeles, CA, USA) (Langfelder and Horvath, 2008).

### 2.5 Immunoscore prediction

Gene expression profile of four immune‐related classes (effector cells, immunosuppressive cells, MHC molecules, and selected immunomodulators) were evaluated to determine the Immunophenoscore* using the available r‐script deposited on GitHub (https://github.com/mui-icbi/Immunophenogram).

### 2.6 Analysis of LncKLHDC7B expression in breast cancer cell lines

MCF10A, BT20, MDA‐MB‐468, MDA‐MB‐231, Hs578‐T, MCF‐7, and HCC1187 cell lines were grown under standard conditions (DMEM or RPMI‐1640 medium, ATCC®, Manassas, VA, USA) according to each line, supplemented with 10% FBS (ATCC®) and incubated at ~ 37 °C with 5% CO_2_. Total RNA was isolated using the Trizol reagent (Invitrogen, Carlsbad, CA, USA) according the manufacturer's recommendation. Subsequently, RNA was quantified by spectrophotometry (Nanodrop) and stored at −80 °C until further processing.

### 2.7 Reverse transcription polymerase chain reaction

Complementary DNA was synthesized using SuperScript III RT‐PCR (Invitrogen) following the manufacturer's recommendations. Briefly, 100 ng of total RNA was used to synthesize cDNA in a final reaction volume of 20 μL. The real time PCR mixture contained 1 μL of cDNA, 5 μL 2× TaqMan Universal Master Mix (Applied Biosystems), 0.5 μL TaqMan® probes (Hs00536653_s1, custom from tcon_00029630), and 3.5 μL of nuclease‐free water. *GAPDH* (Hs99999905_m1) and *U87* (Hs03298717_s1) were used as endogenous controls for coding and non‐coding genes, respectively. The fold change for each gene (*KLHDC7B* or *LncKLHDC7B*) in treated cells relative to control cells was calculated using the Ct (2^‐ΔΔCT) method, where ΔΔ*C*_t_ = Δ*C*_t_ treated cell (shRNA‐1 or ‐2) – Δ*C*_t_ control cell (NC); Δ*C*_t_ = *C*_t_ coding gene – *C*_t_ *GAPDH* or Δ*C*_t_ = Ct non‐coding gene – *C*_t_ *U87*.

### 2.8 Long non‐coding RNA down‐regulation with shRNA

Short hairpin RNA were generated using the BLOCK‐iT™ U6 RNAi Entry Vector Kit (Invitrogen) following the manufacturer's instructions. Briefly, pairs of cDNA oligos were designed containing four nucleotide overhangs necessary for directional cloning. We generated the double‐stranded oligo (ds‐oligo) and subsequently performed the ligation reaction of ds‐oligo into the pENTR™/U6 vector, which was used to transform competent *Escherichia coli* One Shot® TOP10 cells. Sanger sequencing was used to corroborate the presence and correct orientations of the ds‐oligo insert. Two shRNA were used to silence *LncKLHDC7B* (shRNA‐1, 5′‐CACCGCCTCAGCCCAAGTCTTAACTCGAAAGTTAAGACTTGGGCTGAGGC‐3′ and shRNA‐2, 5′‐CACCGCCCAAGTCTTAACTTCAGCTCGAAAGCTGAAGTTAAGACTTGGGC‐3′) and negative control (NC, 5′‐CACCGGAATTACGGAGTCTTCTTCGCGAACGAAGAAGACTCCGTAATTCC‐3′), with a sequence that does not target any mRNA in the human genome *GFP*.

Silencing of *LncKLHDC7B* was performed in three cell lines: HCC1187 (TNBC), MCF‐7 (Luminal A) and BT‐20 (TNBC) using two shRNA (shRNA‐1 and shRNA‐2), which were transfected using Xfect™ Transfection Reagent Protocol (Clontech, Palo Alto, CA, USA). Briefly, HCC1187 cells (6.5 × 10^5^ cells per well), BT‐20 (5 × 10^5^ cells per well) and MCF‐7 (5 × 10^5^ cells per well) were seeded in 6‐well plates and 3 μg of plasmid was transfected during 24 h. Nanoparticle complexes were removed and replaced with complete growth medium. After 48 h post‐transfection, expression of *LncKLHDC7B* was evaluated by RT‐PCR. The RNA was extracted using Trizol according the manufacturer's recommendations and stored at −80 °C until processing. A > 70% decrease of lncRNA expression was achieved after 48 h post‐transfection using 2–3.5 μg of plasmid. The experiments were carried out in triplicate. The effect of silencing *LncKLHDC7B* on the whole‐genome transcriptional landscape in HCC1187 was analyzed with the HTA 2.0 microarray (Affymetrix; Data accessibility GEO: GSE114468), as described above. Genes with a fold change of > 2 and < −2, and a *P* value <0.05 were considered significant and selected for biological pathway analysis using Ingenuity Pathway Analysis (ipa) software and genetrail2 (Stockel *et al*., 2016).

### 2.9 Migration and invasion assay

Cell migration and invasion assays were performed using Transwell migration chambers with 8‐μm pores (Corning, NY, USA). Transfected cells were harvested, re‐suspended in RPMI without FBS at a concentration of 5 × 10^4^ cells in 100 μL, and seeded into the upper chamber of the 24‐well plate. The lower chamber was filled with 600 μL RPMI containing 10% FBS. For the invasion assay, 1 × 10^5^ cells were plated on chambers precoated with 1.6 mg Matrigel (Corning). Cells were incubated (HCC1187 and MCF‐7 for 24 h, BT‐20 for 6 h) for migration assay and (HCC1187 for 36 h, BT‐20 and MCF‐7 for 48 h) for invasion assay at 37 °C, in 5% CO_2_. Each experiment was done in triplicate. At the end of the experiments, cells that migrated to the reverse side of the Transwell membrane were fixed with paraformaldehyde (3.7%), stained with crystal violet, and counted using imagej software (Madison, WI, USA) (Schneider *et al*., 2012).

### 2.10 Apoptosis assay

Apoptosis was induced with ~ 50 μm of 2‐amino‐N‐quinolin‐8‐yl‐benzenesulfonamide (QBS; A3105, Sigma‐Aldrich, St. Louis, MO, USA) for 24 h before the end of 48 h post‐transfection. The cells were then immediately harvested by trypsinization and washed with PBS. The cells were re‐suspended in binding buffer and stained with Annexin V and PI (FITC Annexin V/ Dead cell Apoptosis kit, Invitrogen) for 15 min in the dark at room temperature. The stained cells were examined by cytometry. The cells were categorized into early apoptotic cells and late apoptotic cells (bounded in red lines).

### 2.11 Statistical analysis

Statistical significance was analyzed using graphpad prism (version 6, San Diego, CA, USA) and stata (Version 12, College Station, TX, USA) software. Variances in Kruskal–Wallis tests, ANOVA test, Student's *t* test, and chi‐square tests were performed for all comparisons involving categorical variables. Correlation between variables was determined by Spearman's correlation coefficient. The Kaplan–Meier method and survival differences among groups were assessed by log‐rank test. A *P* value < 0.05 was considered significant (*P <* *0.05, **P <* *0.01, ***P <* *0.001).

## 3 Results

### 3.1 Population characteristics and TNBC subtypes distribution

A total of 156 samples derived from patients with TNBC were included in this study. The mean age of patients was 53 years old (range 26–88) and infiltrating ductal carcinoma was the most frequent type of tumor. Clinical and pathological information on TNBC patients included in this study is shown in Table 1. TNBC subtypes were identified using the TNBCtype algorithm, which assigned samples to the six triple‐negative breast cancer subtypes. Analyzing our TNBC cohort, the majority of tumors were clustered as IM and M (22.4%, *n* = 35/156), followed by BL1 (14.7%, *n* = 23/156) and LAR subtypes (12.2%, *n* = 19/156), BL2 (7.7%, *n* = 12/156), MSL subtype (7%, *n* = 11/156), and UNS (13.5%, *n* = 21/156) (Fig. S1a). The distribution of the TNBC subtypes in our cohort was similar to previous studies (Lehmann *et al*., 2011; Liu *et al*., 2016; Masuda *et al*., 2013) (Fig. S1b). david enrichment analysis (Huang da *et al*., 2009) was applied to each of the TNBC subtypes to corroborate previously reported signaling pathways enriched in each TNBC subtype (Table S1, Fig. S2).

**Table 1:** Clinical‐pathological characteristics of the population

Characteristics	Number	BL1	BL2	IM	M	MSL	LAR	UNS	*P‐*value^a^
	(Total = 156)	(*n* = 23)	(*n* = 12)	(*n* = 35)	(*n* = 35)	(*n* = 11)	(*n* = 19)	(*n* = 21)	
Age (Mean ± SD)	145	47.7**±**10.4	59**±**14.6	50.1**±**12.6	55.2**±**14.2	53.3±10.4	59.1±15.6	51.9±14.6	0.0835^b^
Tumor grade									
≤II	26	1 (3.8)	1 (2.1)	3 (5.5)	7 (5.9)	1 (1.9)	8 (3.1)	5 (3.6)	
>II	110	19 (16.2)	10 (8.9)	26 (23.5)	24 (25.1)	9 (8.1)	8 (12.9)	14 (15.4)	**0.0148** c
Unknown	20								
Tumor size (cm)									
≤ 2 cm	64	6 (9.3)	6 (5.1)	12 (14.4)	15 (13.9)	7 (4.6)	9 (7.4)	9 (9.3)	
> 2 cm	74	14 (10.7)	5 (5.9)	19 (16.6)	15 (16.1)	3 (5.4)	7 (8.6)	11 (10.7)	0.398^c^
Unknown	18								
Type histology									
IDC	117	17 (15.9)	6 (9.2)	29 (26.7)	28 (26.7)	8 (8.4)	13 (13.4)	16 (16.7)	
Other	23	2 (3.1)	5 (1.8)	3 (5.3)	4 (5.3)	2 (1.6)	3 (2.6)	4 (3.3)	0.168^c^
Unknown	16								
Follow up, month (mean)	55.8	50.5	56.6	55	48	65.8	59.8	62.1	0.562^d^
CI	50.7–60.9	34.8–66.2	37.3–75.8	43.9–66.1	37.6–58.4	41.6–90.1	42–77.6	49.2–75.1	
Dead event									
Yes	30	8 (4.2)	4 (2.3)	9 (6.8)	5 (6.8)	1 (2.3)	1 (3.4)	2 (4.2)	0.064^c^
No	112	12 (15.8)	7 (8.7)	23 (25.2)	27 (25.2)	10 (8.7)	15 (12.6)	18 (15.8)	
Unknown	14								

BL1, basal‐like 1; BL2, basal‐like 2; CI, 95% confidence interval; IDC, infiltrating ductal carcinoma; IM, immunomodulatory; LAR, luminal androgen receptor; M, mesenchymal; MSL, mesenchymal stem‐like; SD, standard deviation; UNS, undetermined.

a Unknown data were not included for the statistical significance.

b ANOVA test.

c Chi‐square test *P* < 0.05 (in bold).

d Kruskal–Wallis test.

### 3.2 Unique lncRNA expression profiles in TNBC subtypes

Once our tumor cohort was classified into TNBC subtypes, we identified expression patterns of lncRNA among the tumor subclasses (Fig. 1A). In particular, 710 lncRNA showed differential expression: 84 were altered in at least two of the TNBC subtypes, whereas 524 were only altered in a particular tumor subtype (Fig. 1B, Tables 2 and S2). Comparison between the LAR subtype and all other subtypes yielded the highest number of differentially expressed lncRNA (50.9%, *n* = 362/710) followed by M subtype (16.5%, *n* = 117/710) and IM subtype (14.5%, *n* = 130/710). The MLS subtype showed 98 altered lncRNA, whereas the basal subtypes (BL1 and BL2) had a lower number of deregulated lncRNA (19 and 11 transcripts, respectively) (Fig. 1B, Tables 2 and S2).

**Table 2:** Up‐regulated and down‐regulated lncRNA with higher rate of change between TNBC subtypes

lncRNA name	Fold change^a^	ANOVA *P*‐value	FDR *P*‐value	TNBC subtype
*RP11‐532E4.2*	16.98	0.000133	0.015499	BL1
*LINC01956*	3.26	6.72E‐13	4.54E‐08	BL1
*LINC01123*	1.88	7.11E‐07	0.000572	BL1
*RP11‐619J20.1*	−1.65	0.000054	0.009449	BL1
*RP11‐815J21.4*	−1.65	0.000489	0.031038	BL1
*RP1‐142L7.5*	−1.82	0.000059	0.00983	BL1
*RP11‐116G8.5*	2.73	0.000015	0.060613	BL2
*RP11‐3K16.2*	2.22	0.000036	0.089263	BL2
*LINC01133*	1.69	0.000655	0.307124	BL2
*LINC02095*	−7.91	0.000565	0.300484	BL2
*RP4‐620F22.2*	9.87	0.00E+00	0.00E+00	IM
*RP5‐1171I10.5*	1.9	1.71E‐14	2.95E‐12	IM
*ELMO1‐AS1*	1.71	7.48E‐10	5.31E‐08	IM
*RP13‐455A7.1*	−1.79	0.000545	0.014289	IM
*CTD‐2033D15.1*	−2.98	0.000041	0.001466	IM
*IFNG‐AS1*	1.82	6.93E‐12	6.63E‐10	IM
*RP11‐206M11.7*	80.48	1.78E‐15	1.08E‐12	LAR
*LINC00993*	4.24	8.76E‐07	0.000048	LAR
*PCAT18*	2.56	1.11E‐16	8.24E‐14	LAR
*AF178030.2*	−7.68	9.13E‐10	1.41E‐07	LAR
*LINC01152*	−2.27	0.000181	0.002271	LAR
*VIM‐AS1*	−1.83	3.63E‐07	0.000024	LAR
*GAS5*	−1.63	0.000023	0.000538	LAR
*LINC02095*	20.31	1.11E‐16	1.50E‐12	M
*RP11‐84E24.2*	7.5	0.00E+00	0.00E+00	M
*SOX9‐AS1*	13.71	0.00E+00	0.00E+00	M
*H19*	2.99	0.000066	0.002324	M
*ANO1‐AS1*	2.13	0.000059	0.00212	M
*CASC15*	1.82	9.47E‐08	0.00001	M
*RP11‐553K8.5*	−3.39	6.12E‐10	2.27E‐07	M
*RP11‐989E6.8*	−2.97	4.34E‐07	0.000037	M
*ANKRD44‐IT1*	−1.86	8.62E‐08	0.00001	M
*MEG3*	4.21	5.29E‐11	4.35E‐08	MSL
*AC133106.2*	3.97	1.11E‐09	6.08E‐07	MSL
*DNM3OS*	3.73	0.000039	0.003966	MSL
*VCAN‐AS1*	3.22	0.000019	0.002127	MSL
*CARMN*	2.44	2.25E‐11	2.03E‐08	MSL
*TCONS_l2_00019097*	−1.59	0.000161	0.0125	MSL
*LINC00302*	−1.64	0.000782	0.042797	MSL
*TCONS_l2_00003601*	−1.89	0.000845	0.045414	MSL

a Results from tac software (Affymetrix).

To validate the lncRNA expression patterns in the TNBC subtypes, we corroborated the expression of some candidates using an independent cohort containing lncRNA profiles. Consistent with our data, a set of lncRNA was differentially expressed in the comparisons between BL1, BL2, M, MSL, IM, and LAR tumors in our *in silico* analysis (Fig. S3). Validation of the expression of this set of lncRNA in an independent cohort suggests that they might play important biological roles in the establishment of TNBC subtypes.

### 3.3 Identifying biological pathways associated with lncRNA in TNBC subtypes

**Figure 2:**
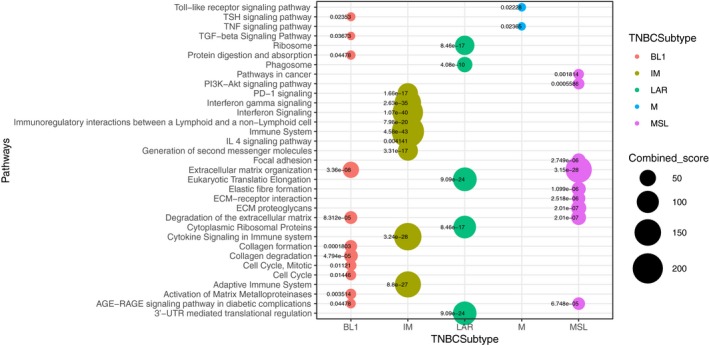
Overview of the biological pathways by guilt‐by‐association analysis across TNBC subtypes by lncRNA‐mRNA co‐expression. Guilt‐by‐association analysis showing the significant enriched pathways of the resulted co‐expression mRNA‐lncRNA. Pathway enrichment analysis resulted in significant association with cancer‐related signaling. The *y*‐axis label represents pathways and the *x*‐axis label represents TNBC subtypes. Bubble chart shows biological pathways enrichment by differential expression of lncRNA for each TNBC subtype. Size and color of the bubble represent the score each pathway and TNBC subtypes, respectively.

To gain insight into the biological relevance of the altered lncRNA, we used a guilt‐by‐association approach to investigate their relationship to dysregulated mRNA and their possible impact on different pathways. This analysis revealed a significant association between some of the differentially expressed lncRNA and mRNA, as well as the enrichment in key breast cancer‐related pathways of associated coding genes for each TNBC subtype. lncRNA altered in BL1 correlated most strongly with extracellular matrix organization, cell cycle and transforming growth factor‐beta (TGF‐β) signaling. IM lncRNA are most strongly related to antigen processing, interferon signaling and immune system. LAR contains lncRNA that are potentially involved in translation regulation. Altered lncRNA in M tumors are associated with toll‐like receptor and TNF signaling. Finally, the co‐expression correlation network of altered lncRNA‐mRNA in the MSL subtype was enriched in focal adhesion, ECM receptor interaction, and PI3K‐Akt signaling pathway (Fig. 2, Table S3). With the exception of the BL2 subtype, where we did not find any significant association, guilt‐by‐association analyses suggested functions for a set of altered lncRNA by their weighted co‐expression with related mRNA that defined a group of pathways with relevant correlations with lncRNA expression portraits in human tumors.

### 3.4 Co‐expression of lncRNA and coding genes in IM subtypes

Long non‐coding RNAs can function as enhancers of the expression of both adjacent and distant genes. To prioritize the most biologically relevant lncRNA in the IM subtypes (one of the most frequent subtypes), as a first approach we identified lncRNA (intergenic or intragenic) with possible regulatory functions over their neighboring genes; thus we analyzed lncRNA–mRNA pairs positively co‐expressed (Fig. S4, Table S4). This approach defined the positive correlation of *KLHDC7B* and *ENSG00000226738*.

### 3.5 *LncKLHDC7B* (*ENSG00000226738*) and *KLHDC7B* show a correlated expression pattern in the immunomodulatory subtype

**Figure 3:**
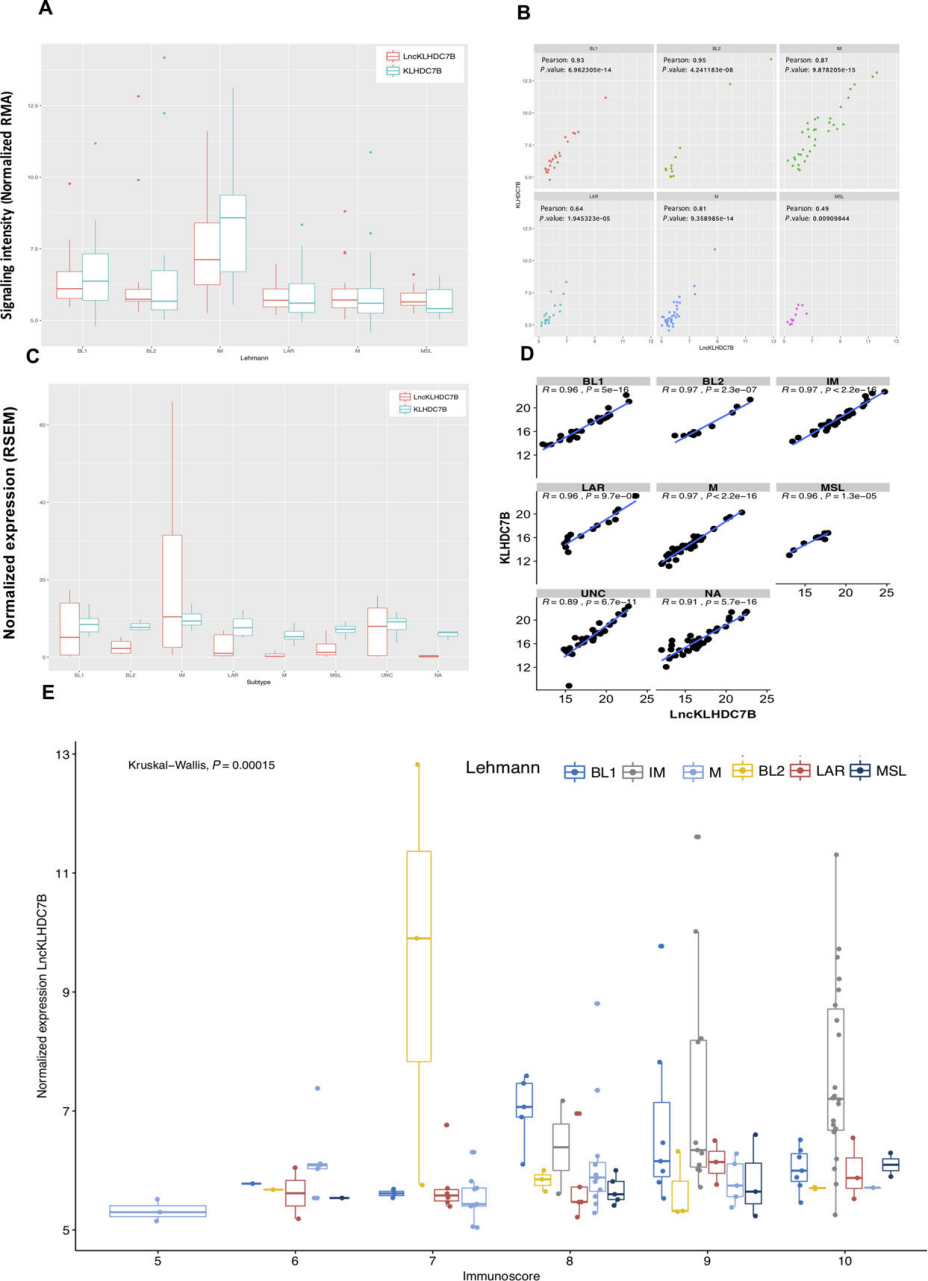
Evaluation of *LncKLHDC7B* and *KLHDC7B* expression in TNBC subtypes. (A) *LncKLHDC7B* and *KLHDC7B* expression levels in our cohort and (C) TCGA database. (B) Correlation between gene expression level of *LncKLHDC7B* and *KLHDC7B* across TNBC subtypes in our dataset, and TCGA database (D). (E) Relation across the immunoscore with the expression of *LncKLHDC7B* in our cohort. Kruskal–Wallis test was performed to determine significance. The *y*‐axis label represents the normalized expression of the *LncKLHDC7B* and the *x*‐axis label represents the immunoscore.

**Figure 4:**
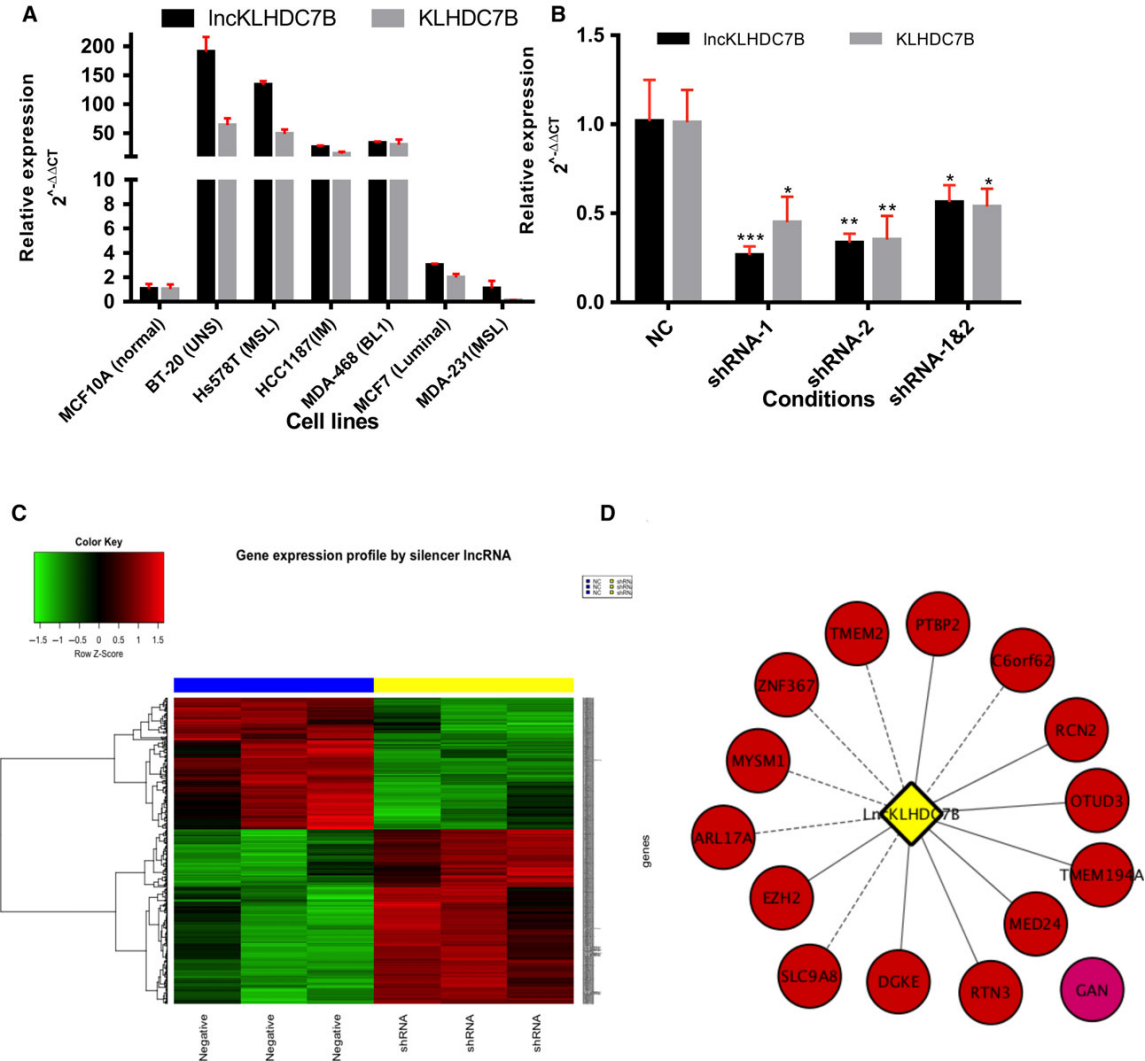
Impact of *LncKLHDC7B* silencing on the TNBC immunomodulatory phenotype. (A) Validation of *LncKLHDC7B* and *KLHDC7B* expression in a panel of breast cancer cell lines. (B) Expression levels of *LncKLHDC7B* in immunomodulatory phenotype (HCC1187) of breast cancer cell line after silencing by shRNA system. *KLHDC7B* coding gen expression is affected by *LncKLHDC7B* silencing. All data are shown as the mean ± SD of at least three independent experiments. Student's *t* test was performed to determine significance *P < 0.05, **P < 0.01, ***P < 0.001 of NC vs shRNA. (C) Heatmap from transcripts altered by *LncKLHDC7B* silenced. The microarray was performed in triplicate for the NC condition (blue bar) and shRNA‐1 (yellow bar). Red represents elevated and green down‐regulated expression. (D) Prediction of interactions between lncRNA and mRNA targets sub‐expressed by the silencing of the *LncKLHDC7B*. Continuous and dotted lines represent a medium and high interaction, respectively.

The lncRNA *ENSG00000226738* (which we call *LncKLHDC7B*) and its coding gene *KLHDC7B* were among one the most correlated pairs evaluated (*r* ^2^ = 0.9, *P* < 0.0001; Figs S4 and 3A–D). The overexpression of the pair *LncKLHDC7B/KLHDC7B* as well as their correlation in the IM subtype was also validated in TCGA data (Fig. 3C,D, Table S4). We evaluated the expression of both transcripts in a panel of breast cancer cell lines, which showed increased expression of both genes in many tumor cell lines, including IM cell line (HCC1187), in contrast to a non‐tumorigenic epithelial cell line MCF10A (Fig. 4A). Interestingly, these observations were reproducible in breast cancer tissues compared with normal tissue using databases from TCGA (Fig. S6). We noticed a distinct expression pattern of *KLHDC7B* and *LncKLHDC7B* among the different triple‐negative subgroups, both in tumors and cell line models (Figs 3A‐D, 4A and S6). To further validate the relationship of *LncKLHDC7B* with the immunophenotype, we calculated the immunophenoscore of our profiled cohort. Our findings showed distinct expression patterns across the scores, where the highest scores (9 and 10) present a significant enrichment of *LncKLHDC7B*, particularly in the IM subtype (Fig. 3E). Given these results, we selected the HCC1187 cell line to investigate further the functional role of *LncKLHDC7B* in the immunomodulatory subtype.

We then chose the *LncKLHDC7B*/*KLHDC7B* pair for further analysis for the following reasons: (1) *LncKLHDC7B* may be a subtype tumor marker of the IM subtype, but its function is not well described in the literature; (2) *LncKLHDC7B* presents a significant association with its coding gene (localized in the same locus), which indicates a possible regulatory activity; (3) our guilt‐by‐association analysis highlighted correlations between the lncRNA and key cancer‐related pathways such as antigen presentation, natural killer‐mediated cytotoxicity, interferon signaling, and cell adhesion (Fig. S5). All these data suggest a potential role for *LncKLHDC7B* in the TNBC subtype establishment and biological regulatory programs.

### 3.6 Silencing of *LncKLHDC7B* down‐regulates *KLHDC7B* and impacts the expression of a large number of genes

We evaluated the role of *LncKLHDC7B* on oncogenic phenotypes in the IM breast cancer HCC1187 cell line model. The knockdown of *LncKLHDC7B* was achieved with ~ 75% silencing efficiency (Fig. 4B) and a 50% inhibition of the expression of *KLHDC7B* gene (Fig. 4B). Furthermore, a complete genomic analysis of the transcriptomic state after silencing of *LncKLHDC7B* revealed an impact on the global expression of 1265 transcripts (Fig. 4C, Table S5), including several transcripts from the Kelch family. These data demonstrated that *LncKLHDC7B* might regulate gene expression both in *cis*, mediating the transcriptional activation of the *KLHDC7B* gene*,* and in *trans*, by regulating distant genes located throughout the genome.

Long non‐coding RNAs show a diversity of functions through diverse mechanisms, interactions with other RNA molecules being some of the most representative. We applied an mRNA‐lncRNA interaction prediction with a nearest‐neighbor method based on thermodynamic parameters using the LncTar tool (Li *et al*., 2015). Genes most significantly down‐regulated upon *LncKLHDC7B* silencing were evaluated. Our results indicate a high and medium direct interaction between the mRNA and *LncKLHDC7B* (ndG value ≤ −0.1 indicates a true predicted interaction that could not be considered a chance event). Thus, their down‐modulation might be a direct consequence of *LncKLHDC7B* knockdown. The regulator‐signaling network mediated by *LncKLHDC7B* in the IM cell line model is described in Fig. 4D and Table S6. This approach provided relevant information to identify valuable lncRNA–mRNA interactions for predicting RNA targets and figuring out the possible biological function of the lncRNA.

### 3.7 *LncKLHDC7B* modulates the expression of genes associated with relevant hallmarks of cancer

**Figure 5:**
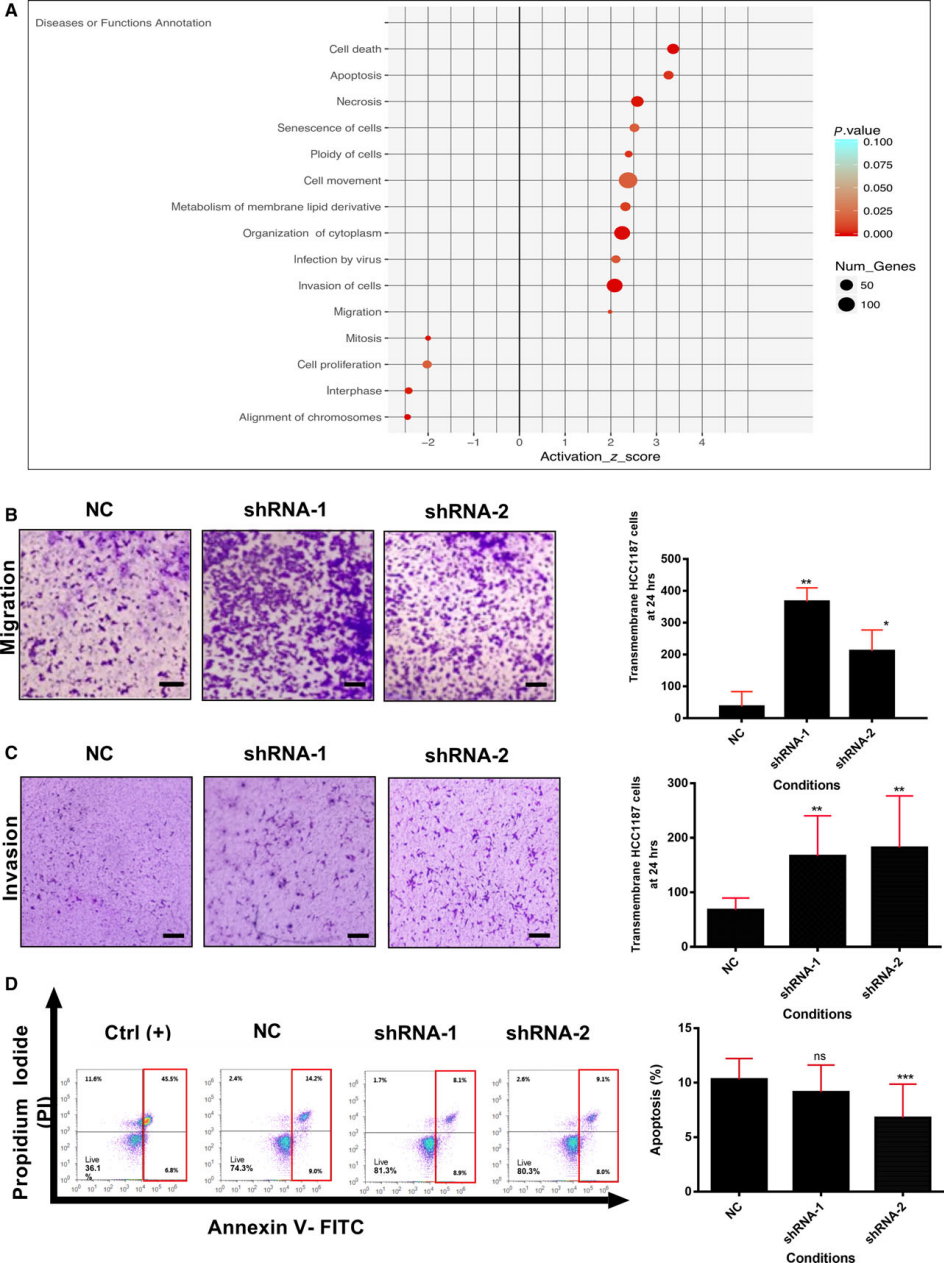
Enrichment and functional analysis for the silencing of *LncKLHDC7B* in HCC1187 cell line. (A) Enrichment of biological process from transcriptional alteration by *LncKLHDC7B* silencing in HCC1187 cell line (IPA analysis). The size of the circle represents the number of genes involved in the biological process; the color indicates the statistical significance. (B) Transwell migration and (C) invasion assay showed that *LncKLHDC7B* silencing increases the migration and invasion of HCC1187. Representative images are shown on the left and quantification on the right. Scale bar: 50 μm. (D) Flow cytometric analysis of apoptosis (early and late) in HCC1187 cell transfected with control and shRNA‐1 and ‐2 after Annexin V/PI staining. All data are shown as the mean ± SD of at least three independent experiments. Student's *t* test was performed to determine significance of NC vs shRNA: *P < 0.05, **P < 0.01, ***P < 0.001.

To investigate the global impact of *LncKLHDC7B* inhibition in the IM cell line model, we performed an Ingenuity Pathway Analysis on up‐ or down‐modulated targets after *LncKLHDC7B* reduction, which revealed a significant over‐representation of cancer‐relevant pathways related to cell migration, cellular death, apoptosis, and invasion (Fig. 5A).

### 3.8 Silencing of *LncKLHDC7B* increases cellular migration and invasion, and decreases cell death

*LncKLHDC7B* knockdown significantly increased the ability of cells to migrate in comparison with control HCC1187 cells (Fig. 5B,C). This oncogenic phenotype could be achieved by the observed up‐modulation of *TNC, CDH3, APP, EPS8,* and *CAV1,* as previously reported (Baek *et al*., 2010; Chen *et al*., 2015a; Diaz *et al*., 2014; Lim *et al*., 2014; Zhang *et al*., 2016). As shown in Fig. 5C, the invasion ability of HCC1187 cells transfected with shRNA was significantly enhanced compared with the control group. This phenotype might be related to the up‐modulation (Table S5) of genes that have been previously involved in the invasion process, such as *MMP2* (Fagan‐Solis *et al*., 2013; Kim and Rhee, 2016), *ADAM9* (Micocci *et al*., 2013), and *AMOT* (Zhang and Fan, 2015). Additionally, the depletion of *LncKLHDC7B* decreased the number of apoptotic cells, reaching ~ 23% of reduction in HCC1187 cells going through apoptotic death (Fig. 5D). This effect might be explained by the down‐regulation of genes related to the induction of apoptosis, such as *PMAIP1* (Zhao *et al*., 2014), *RAD21* (Chen *et al*., 2002; Pati *et al*., 2002), and CCAR2 (known as *DBC1*) (Kim *et al*., 2008), that we observed to be under‐expressed after *LncKLHDC7B* silencing (Table S5).

**Figure 6:**
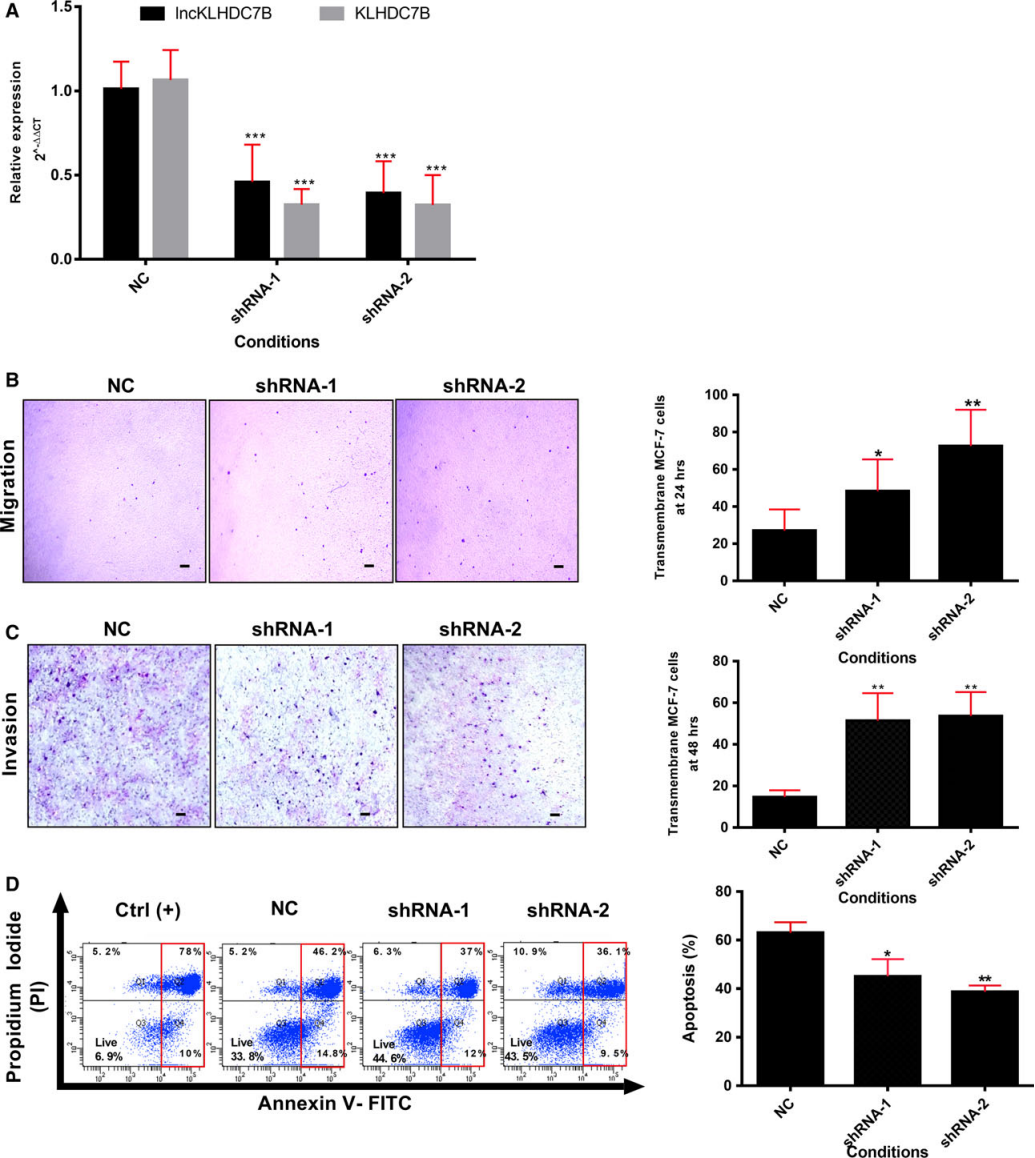
The silencing of *LncKLHDC7B* modulates breast cancer cell migration and invasion as well as resistance to apoptosis in MCF‐7. (A) The expression of *LncKLHDC7B* and *KLHDC7B* in MCF‐7 cells transfected with NC or shRNA‐1 or ‐2 was determined by qRT‐PCR. (B) Transwell migration and (C) invasion assay showed that *LncKLHDC7B* silencing increases the migration and invasion of MCF‐7. Representative images are shown on the left and quantification on the right. Scale bar: 25 μm. (D) Flow cytometric analysis of apoptosis (early and late) in MCF‐7 cell transfected with control and shRNA‐1 and ‐2 after Annexin V/PI staining. All data are shown as the mean ± SD of at least three independent experiments. Student's *t* test was performed to determine significance of NC vs shRNA: *P < 0.05, **P < 0.01, ***P < 0.001.

To determine whether the silencing effect of *LncKLHDC7B* on cellular migration, invasion, and apoptosis events are exclusive for triple‐negative breast cancer, we silenced lncRNA in two additional cell lines (BT‐20 and MCF‐7). Interestingly, the down‐regulation of *LncKLHDC7B* in the MCF‐7 luminal cell line affected *KLHDC7B* mRNA expression (Fig. 6A). Moreover, silencing promoted migration and invasion (Fig. 6B,C) of MCF‐7 cells, as well as a resistance to apoptosis (18–24%), compared with the control group (Fig. 6D).

However, in the triple‐negative cell line BT‐20, we were only able to obtain a 35% of inhibition of *LncKLHDC7B*, and we only observed a 34% down‐regulation of *KLHDC7B* (Fig. S7a,b). In addition, the migration, invasion, and resistance to apoptosis were enhanced, albeit non‐significantly, by an shRNA (shRNA‐1) that had the greatest effect on the silencing the lncRNA in this particular cell line (Fig. S7b‐d).

### 3.9 Clinical implications of *LncKLHDC7B* and *KLHDC7B* expression in triple‐negative breast cancer

**Figure 7:**
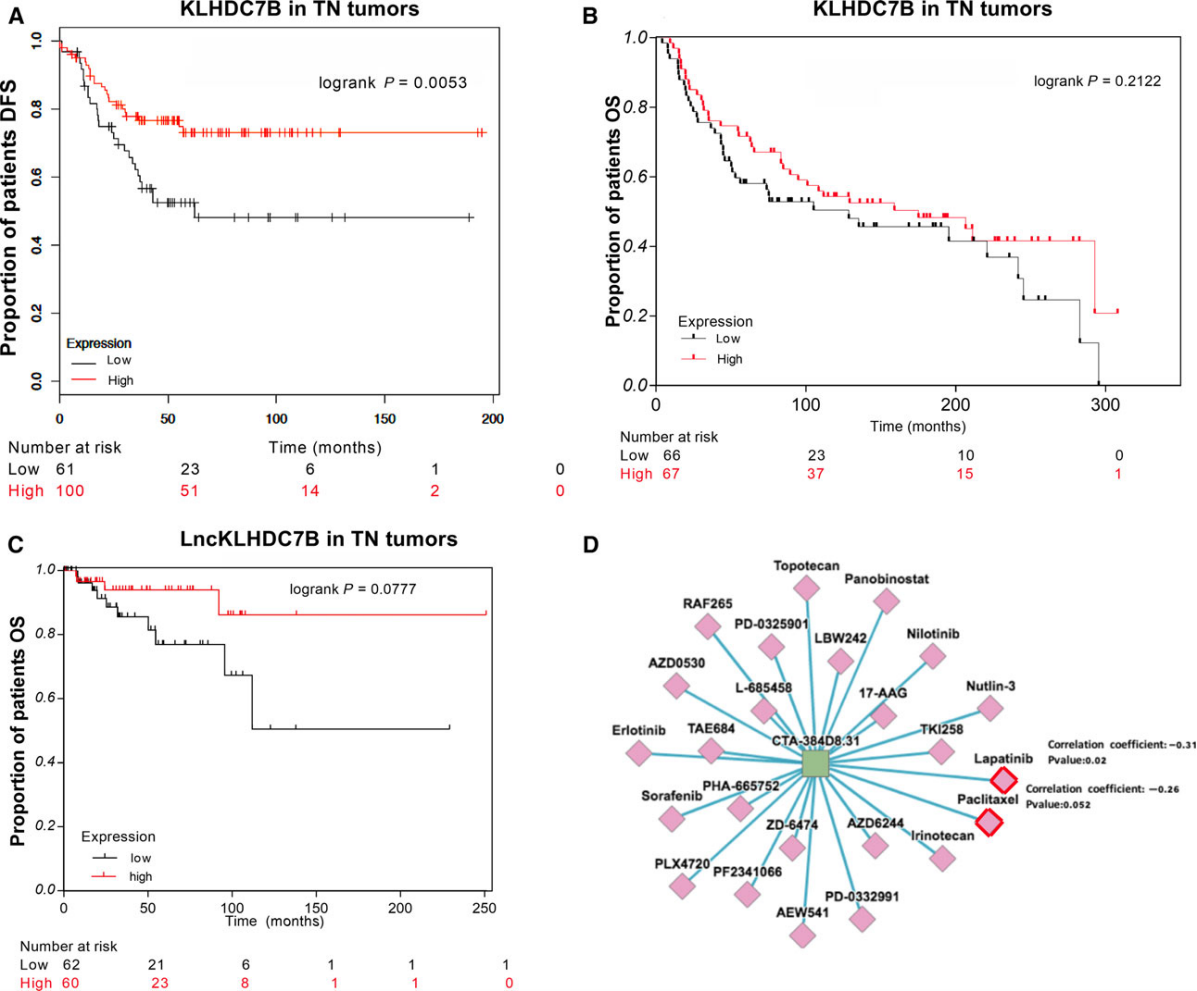
Clinical implications of the sub‐expression of *LncKLHDC7B* and *KLHDC7B* in TNBC. (A) Kaplan–Meier analysis curve of the disease‐free survival (DFS) of TNBC according to *KLHDC7B* expression. (B) Kaplan–Meier analysis of overall survival (OS) according to *KLHDC7B* expression. (C) Kaplan–Meier analysis of prognostic relevance of *LncKLHDC7B* expression from public data. (D) *In silico* prediction of susceptibility to USFDA‐approved treatment drugs related with *LncLHDC7B* expression in triple‐negative breast cancer cell line.

To investigate the potential clinical implication of down‐regulating *LncKLHDC7B* and *KLHDC7B* in triple‐negative breast cancer, we identified and analyzed public data (Anaya, 2016; Curtis *et al*., 2012; Lanczky *et al*., 2016) with available mRNA or lncRNA expression profiles and clinical information (disease‐free survival or overall survival). The down‐modulation of *KLHDC7B* was associated with an increased probability of a recurrent or metastatic event (*n* = 161, *P* = 0.0053, Fig. 7A), but not with risk of death (*n* = 133, *P* = 0.212; Fig. 7B) compared with samples showing a higher expression of the gene. We did not detect a significant probability of death in tumors with low expression of the lncRNA in triple‐negative breast cancer samples (Fig. 7C, but a generalized analysis in breast cancer showed that the low *KLHDC7B* and *LncKLHDC7B* expression was significantly associated with lower survival (HR 1.910, 95% CI 1.147–3.233, log‐rank test, *P* = 0.0137 and HR 1.933, 95% CI 1.161–3.273, log‐rank test, *P* = 0.0120, respectively) compared with patients who presented higher expression of both transcripts (Fig. S8).

Previous studies and our results shown above indicated that lncRNA is differentially expressed between TN breast cancer subtypes, thus lncRNA may be explored as a new class of clinical biomarkers for therapy response or targets for drug discovery (Arun *et al*., 2018; Prabhakar *et al*., 2017; Wu and Du, 2017). To identify drug‐related lncRNA in breast cancer, we explored the association of the *LncKLHDC7B* levels and drug activity with the lncMAP tool (Li *et al*., 2018) (http://bio-bigdata.hrbmu.edu.cn/LncMAP/index.jsp), which computes the Spearman correlation coefficient between lncRNA expression and the half maximal inhibitory concentration (IC_50_) values of 24 drugs approved for oncology purposes in TNBC cell lines. We observed a significant negative correlation between the expression of *LncKLHDC7B* and concentrations of lapatinib and paclitaxel (Fig. 7D), which are being evaluated in various clinical trials (https://clinicaltrials.gov/ct2/home).

## 4 Discussion

Triple‐negative breast cancer is a heterogeneous disease at the cellular, molecular, and clinical levels (Lehmann *et al*., 2011). Long non‐coding RNA have recently emerged as important players implicated in relevant biological processes (Martin and Chang, 2012) as well as potential cancer biomarkers or therapeutic targets (Arun *et al*., 2018; Chandra Gupta and Nandan Tripathi, 2017; Prabhakar *et al*., 2017; Wan *et al*., 2016; Wu and Du, 2017). In the present study, we classified TNBC using mRNA expression profiles (Chen *et al*., 2012) and identified several lncRNA expressed in specific tumor subtypes. The most frequent subtypes in our sample collection were mesenchymal‐like and immunomodulatory, consistent with previous reports (Lehmann *et al*., 2011; Liu *et al*., 2016; Masuda *et al*., 2013). Regarding tumor subgrouping and clinical characteristics, we did not detect any significant association between TNBC subtypes and tumor size, histology or survival data, except for tumor grade.

A class comparison analysis identified a profile of 710 differentially expressed lncRNA among the 156 samples belonging to different TNBC subtypes. A guilt‐by‐association analysis revealed the possible biological pathways associated with the lncRNA expressed for each of the TNBC subtypes except for BL2, possibly due to lack of correlation between the expression of mRNA and lncRNA.

Overall, our findings indicate that lncRNA expression patterns can distinguish TNBC subtypes and that lncRNA might be involved in important pathways related to tumor biology, contributing to the regulatory circuits between lncRNA and mRNA. There was a quite interesting relationship between lncRNA and the immunomodulatory subtype, one of the altered tumor groups in the context of lncRNA transcriptional landscape, in which lncRNA activity seems to impact an important number of pathways involved in the establishment of the immune infiltrated phenotype. Given these results, we decided to further characterize the IM subtype.

In the IM subtype, *LncKLHDC7B* and *KLHDC7B* were up‐regulated with positive correlated expression (*r* ^2^ = 0.9, *P* < 0.0001; Fig. S4), suggesting that the antisense lncRNA might regulate the expression of the *KLHDC7B* coding gene.

Several lines of evidence indicated the importance of the *LncKLHDC7B*/*KLHDC7B* pair of transcripts, including that *LncKLHDC7B* expression is specific for the IM subtype (or, based on our analyses, on breast cancer in a generalized way). *LncKLHDC7B* expression levels are significantly correlated with its coding gene (localized in the same locus) and our guilt‐by‐association analysis highlighted a correlation between lncRNA and key cancer‐related pathways.

*KLHDC7B* (Kelch Domain Containing 7B) is a protein member of the Kelch superfamily, proteins related to cellular processes such as cytoskeletal rearrangement and protein degradation (Adams *et al*., 2000). Alterations in this protein superfamily have been associated with various types of cancer, including leukemia, lung, prostate, brain, and Hodgkin's disease (Gupta and Beggs, 2014). To the best of our knowledge, the only report that associates this gene with breast cancer, reported the hypermethylation and over‐regulation of this gene in mammary tumors, suggesting a possible role as an epigenetic marker (Kim *et al*., 2010). However, the role of its non‐coding antisense *LncKLHDC7B* was unknown.

*LncKLHDC7B* is a 260‐base transcript and has two exons. At the genomic level *LncKLHDC7B* is an antisense lncRNA of ~ 1.26 Kb, located on the long arm of chromosome 22q13.33, sharing a locus with the *KLHDC7B* gene (Kent *et al*., 2002; Volders *et al*., 2015). Different approaches have demonstrated the potential role of lncRNA as regulators of the expression of genes in *cis* or in *trans* (Engreitz *et al*., 2016; Gupta *et al*., 2010). We first corroborated the positive correlation in the expression of *LncKLHDC7B* and *KLHDC7B* in independent tumor datasets and in cell lines. This over‐expression was corroborated in the immunomodulatory HCC1187 cell line. Subsequently, we silenced the *LncKLHDC7B* using shRNA, which resulted in the repression of the *KLHDC7B* coding gene. Thus, *LncKLHDC7B* might be acting as a *cis* transcriptional regulator of its coding gene *KLHDC7B*.

We also analyzed the effects of *LncKLHDC7B* knockdown on the global transcriptional landscape in HCC11887 cells. We found that expression of several genes was modified after silencing the lncRNA, suggesting its potential role as a transcriptional regulator in *trans*. Enrichment pathways analysis identified a significant up‐modulation of cell death, necrosis, apoptosis, cell movement, migration, invasion, and organization of cytoplasm upon lncRNA silencing. Interestingly, we observed the deregulation of several genes of the Kelch family both upwards and downwards, some of them related to processes such as cell migration and invasion, as well as apoptosis (Lian *et al*., 2016; Ohta *et al*., 2010).

Previous studies showed that silencing *KLHL39*, another member of the Kelch family, increased cell migration and invasion, and decreased cell death by anoikis (Chen *et al*., 2015b). In accordance with these data, we showed that silencing *LncKLHDC7B* significantly increased the cellular migration and invasion, as well as the resistance to apoptosis in HCC1187 and MCF‐7 cells. These observations are also supported by our analysis of the BT‐20 cell line (which showed higher expression of *LncKLHDC7B* and *KLHDC7B*), where a ~ 30% silencing was obtained by one of the shRNA, although in a non‐significant way, of migration and cellular invasion with a tendency toward resistance to apoptosis. In general, these observations suggest that the effect of *LncKLHDC7B* and *KLHDC7B* inhibition might be applicable beyond the triple‐negative subtype.

Previous studies suggest that the overexpression of protein members of the Kelch family, such as *KLHL2,* increases apoptosis (Tseng and Bixby, 2011) or, in the case of silencing *KLHDC7B,* favors death resistance, as previously published (Jeong *et al*., 2018). Here, we show that the silencing of *LncKLHDC7B* led to resistance to cell death. Similarly, it has been reported that silencing of members of the Kelch family leads to death resistance by anoikis and is associated with lower survival in colorectal cancer (Chen *et al*., 2015b).

These results support the potential role of *LncKLHDC7B* as a gene expression regulator of *KLHDC7B*, and its association with cytoskeletal rearrangement, cell migration, and invasion. Further evidence of the potential role of the *LncKLHDC7B*–*KLHDC7B* correlated expression in cancer comes from the recent description of a novel 5′ alternative splicing site in the *KLHDC7B* gene in cervical squamous cell carcinoma. This novel splice site coincides with the location of a lncRNA (Tcons_00029745) and was related to cellular differentiation and tumor size in this disease (Guo *et al*., 2015).

Our data indicate that *LncKLHDC7B* is required for cell migration and invasion inhibition, and contributes to apoptosis induction, suggesting its important regulatory activity in TNBC tumors with enrichment of *LncKLHDC7B,* or maybe a generalized process in breast cancer.

To explore further the altered expression of *LncKLHDC7B* and *KLHDC7B* and their clinical implication in breast cancer, we evaluated the correlation between their expression and survival probability in a public database. Down‐regulation of *KLHDC7B* was associated with lower disease‐free survival and *LncKLHDC7B* was closely associated with poor clinical outcome in triple‐negative tumors and breast cancer in general. As far as we know, this is the first report of the association of *KLHDC7B* or *LncKLHDC7B* expression with poor prognosis in breast cancer. In addition, we analyzed the clinical implication of *LncKLHDC7B* expression and oncological drugs. Our results suggest a higher sensitivity to lapatinib and paclitaxel in cells over‐expressing the *LncKLHDC7B*; however, the limitation of this approach was the lack of *in vitro* validation.

## 5 Conclusions

We explored the lncRNA expression landscape of the very complex and heterogeneous disease represented by triple‐negative breast cancer, and identified several lncRNA with subtype‐specific expression. Through this analysis we unveiled a correlation in the expression of diverse lncRNA with their corresponding coding genes in the IM subtype, including *LncKLHDC7B*‐*KLHDC7B*. Silencing of *LncKLHDC7B* led to altered expression of *KLHDC7B,* a member of the Kelch family, and increased cell migration and invasion with decreased apoptosis, indicating that *LncKLHDC7B* may modulate the aggressiveness of breast cancer IM subtype at least in part by regulating the expression of its host gene *KLHDC7B*. We observed a consistent trend toward shorter survival for patients with low expression levels of *LncKLHDC7B* and *KLHDC7B*. Although the clinical relevance of *LncKLHDC7B* expression remains incompletely understood, our results reaffirm the down‐modulation of these transcripts in patients with aggressive clinical TN tumors and suggest their association with poor outcomes. These data must be further characterized to elucidate the exact mechanism involved in these observations.

## Author contributions

B.A.: performed most of the experimental work and data analysis. R.C., C.T., R.R.: data analysis and discussion. R.V., A.R., J.M., R.G.: sample collection and processing. A.O.: data analysis. B.P.: histopathological review of the cases. D.R., V.C., T.T.: patient identification and clinical follow up. T.E., I.M., B.A., H.M.: project coordination and leadership, data analysis, and study design. All authors contributed to the writing of the manuscript.

## Conflict of interest

A.H.M. has received grants from Astra Zeneca, but these are not related to this particular project. All other authors declare no conflict of interest.

## Supporting information


**Table S1.** Gene ontology and KEGG pathway enrichment analysis from differentially expressed genes from TNBC subtypes.

**Table S2.** Differentially expressed lncRNA in the TNBC subtypes.

**Table S3.** *In silico* prediction of biological pathways by guilt‐by‐association analysis across lncRNA‐mRNA co‐expressed in the TNBC subtypes.

**Table S4.** Correlation analysis of coding and non‐coding genes co‐expressed positively in the immunomodulatory phenotype.

**Table S5.** Differentially expressed genes that are significantly modulated after *lncKLHDC7B* silencing in HCC1187 cells.

**Table S6.** Prediction lncRNA‐mRNA interactions from down‐regulated genes after silencing *lncKLHDC7B*.

**Fig. S1.** (a) Frequency of TNBC subtypes in this study and (b) other studies (Cancer Genome Atlas, 2012; Curtis *et al*., 2012; Lehmann *et al*., 2011; Liu *et al*., 2016; Masuda *et al*., 2013).
**Fig. S2.** Gene ontology from FFPE TNBC samples in this study.
**Fig. S3.** Independent validation of some LncRNA in TNBC subtypes.
**Fig. S4**. Correlation analysis of coding and non‐coding genes co‐expressed positively in the immunomodulatory phenotype.
**Fig. S5.** Guilt‐by‐association analysis.
**Fig. S6.** Up‐modulation of LncKLHDC7B and KLHDC7B in tumor samples of breast cancer.
**Fig. S7.** Functional analysis by the silencing of *LncKLHDC7B* in BT‐20 cell line.
**Fig. S8.** Kaplan–Meier curve of overall survival (OS).
