# Supplementary material for: Expression of long non‐coding RNA ENSG00000226738 (LncKLHDC7B) is enriched in the immunomodulatory triple‐negative breast cancer subtype and its alteration promotes cell migration, invasion, and resistance to cell death
Source: Mol Oncol. 2019 Feb 9;13(4):909–27. doi: 10.1002/1878-0261.12446 (PMC6441920; doi:10.1002/1878-0261.12446)
Supplement: Supplementary file 7 — Fig. S1. (a) Frequency of TNBC subtypes in this study and (b) other studies (Cancer Genome Atlas, 2012; Curtis et al., 2012; Lehmann et al., 2011; Liu et al., 2016; Masuda et al., 2013). Fig. S2. Gene ontology from FFPE TNBC samples in this study. Fig. S3. Independent validation of some LncRNA in TNBC subtypes. Fig. S4. Correlation analysis of coding and non‐coding genes co‐expressed positively in the immunomodulatory phenotype. Fig. S5. Guilt‐by‐association analysis. Fig. S6. Up‐modulation of LncKLHDC7B and KLHDC7B in tumor samples of breast cancer. Fig. S7. Functional analysis by the silencing of LncKLHDC7B in BT‐20 cell line. Fig. S8. Kaplan–Meier curve of overall survival (OS). [file MOL2-13-909-s007.pdf]

Supplementary Information

The file contains

- Supplementary information figure1-8

Supplementary Information

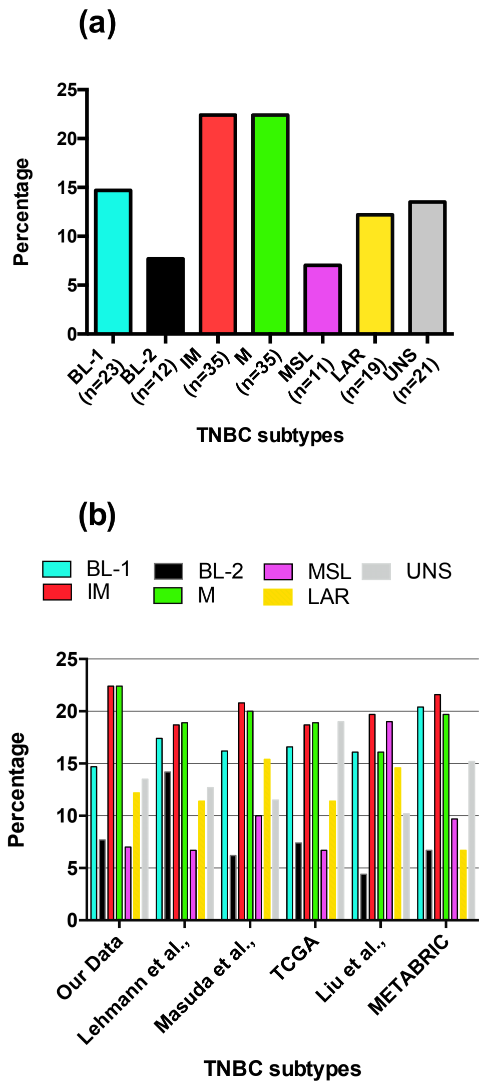

Supplementary figure 1. (a) Frequency of TNBC subtypes in this study and (b) other studies (Cancer Genome Atlas, 2012; Curtis et al., 2012; Lehmann et al., 2011; Liu et al., 2016; Masuda et al., 2013 .

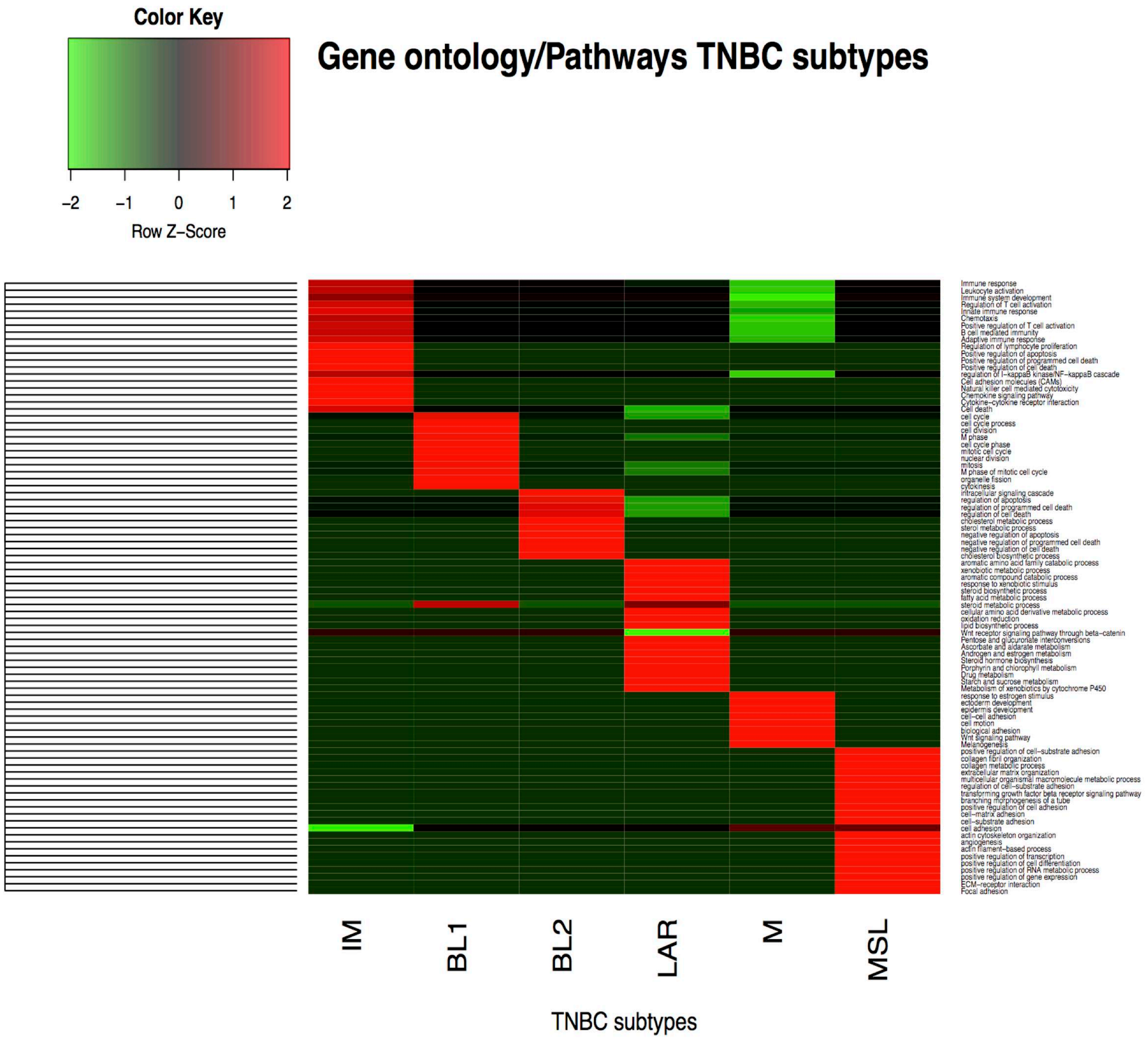

Supplementary figure 2. Gene ontology from FFPE TNBC samples in this study. Each subtype shows biological enrichment according to gene expression profile from DAVID analysis.

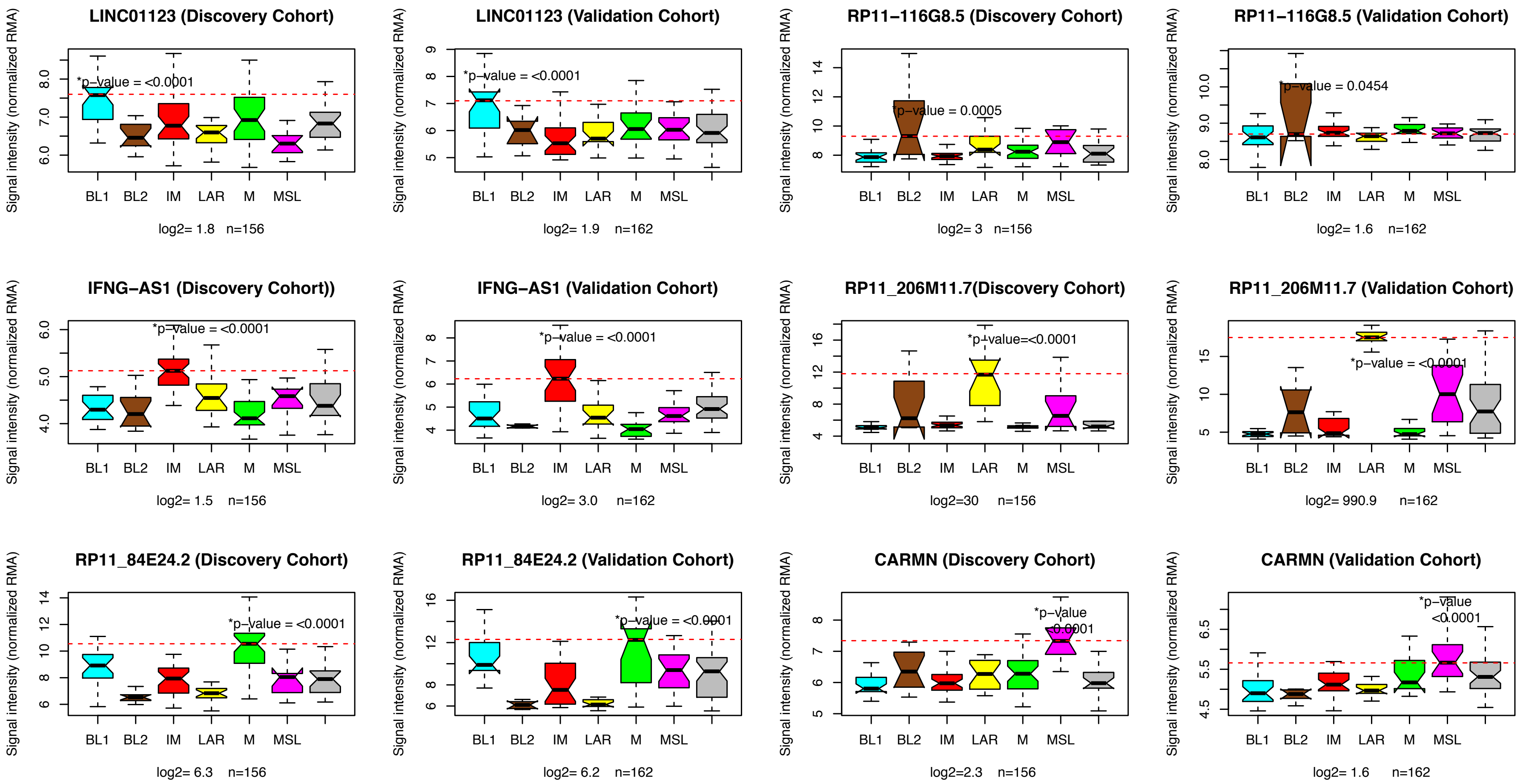

Supplementary figure 3. Independent validation of some LncRNAs in TNBC subtypes. The plot represent levels of normalized fluorescence intensity (RMA) of LncRNAs evaluated in our cohort (n=156) and in the validation cohort (n=160) (GEO: GSE76250). FC: fold change. The dotted line shows the mean expression. BL1: Basal-like 1, BL2: Basal-like 2, IM: immunomodulatory, M: mesenchymal, MSL: mesenchymal stem-like, LAR: luminal androgen receptor.

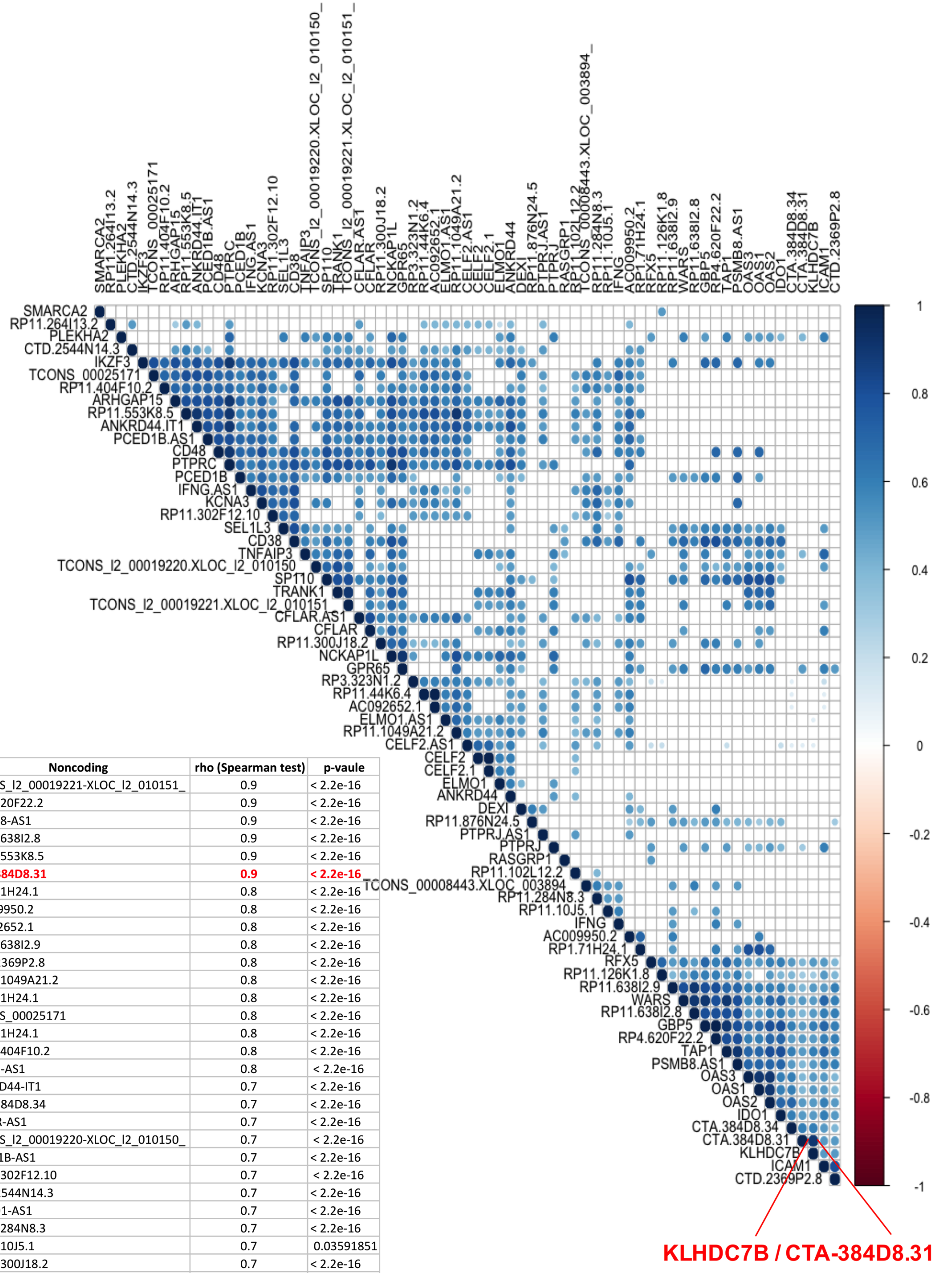

Coding	Noncoding	rho (Spearman test)	p-vaule
TRANK1	TCONS_l2_00019221-XLOC_l2_010151_	0.9	< 2.2e-16
GBP5	RP4-620F22.2	0.9	< 2.2e-16
TAP1	PSMB8-AS1	0.9	< 2.2e-16
WARS	RP11-638I2.8	0.9	< 2.2e-16
PTPRC	RP11-553K8.5	0.9	< 2.2e-16
**KLHDC7B**	**CTA-384D8.31**	**0.9**	**< 2.2e-16**
OAS1	RP1-71H24.1	0.8	< 2.2e-16
SP110	AC009950.2	0.8	< 2.2e-16
ARHGAP15	AC092652.1	0.8	< 2.2e-16
WARS	RP11-638I2.9	0.8	< 2.2e-16
ICAM1	CTD-2369P2.8	0.8	< 2.2e-16
NCKAP1L	RP11-1049A21.2	0.8	< 2.2e-16
OAS3	RP1-71H24.1	0.8	< 2.2e-16
IKZF3	TCONS_00025171	0.8	< 2.2e-16
OAS2	RP1-71H24.1	0.8	< 2.2e-16
CD48	RP11-404F10.2	0.8	< 2.2e-16
CELF2	CELF2-AS1	0.8	< 2.2e-16
ANKRD44	ANKRD44-IT1	0.7	< 2.2e-16
KLHDC7B	CTA-384D8.34	0.7	< 2.2e-16
CFLAR	CFLAR-AS1	0.7	< 2.2e-16
TRANK1	TCONS_l2_00019220-XLOC_l2_010150_	0.7	< 2.2e-16
PCED1B	PCED1B-AS1	0.7	< 2.2e-16
SEL1L3	RP11-302F12.10	0.7	< 2.2e-16
PLEKHA2	CTD-2544N14.3	0.7	< 2.2e-16
ELMO1	ELMO1-AS1	0.7	< 2.2e-16
KCNA3	RP11-284N8.3	0.7	< 2.2e-16
TNFAIP3	RP11-10J5.1	0.7	0.03591851
GPR65	RP11-300J18.2	0.7	< 2.2e-16
RFX5	RP11-126K1.8	0.6	< 2.2e-16
IFNG	IFNG-AS1	0.6	< 2.2e-16
CD38	TCONS_00008443-XLOC_003894_	0.6	< 2.2e-16
RASGRP1	RP11-102L12.2	0.6	< 2.2e-16
CELF2	RP3-323N1.2	0.6	< 2.2e-16
DEXI	RP11-876N24.5	0.5	< 2.2e-16
SMARCA2	RP11-264I13.2	0.4	6.71E-08
PTPRJ	PTPRJ-AS1	0.4	9.70E-08
IDO1	RP11-44K6.4	0.4	7.26E-06

Supplementary figure 4. Correlation analysis of coding and non-coding genes co-expressed positively in the immunomodulatory phenotype. In the table shows the coding and long non-coding genes (adjacent or overlapping) from IM subtype TNBC. Triangular heat map representing the correlation coefficients matrix of coding and non-coding genes co-expressed, the blue dots indicate the statistically significant positive correlations (p<0.0001), and blank squares indicate non-significant correlations.

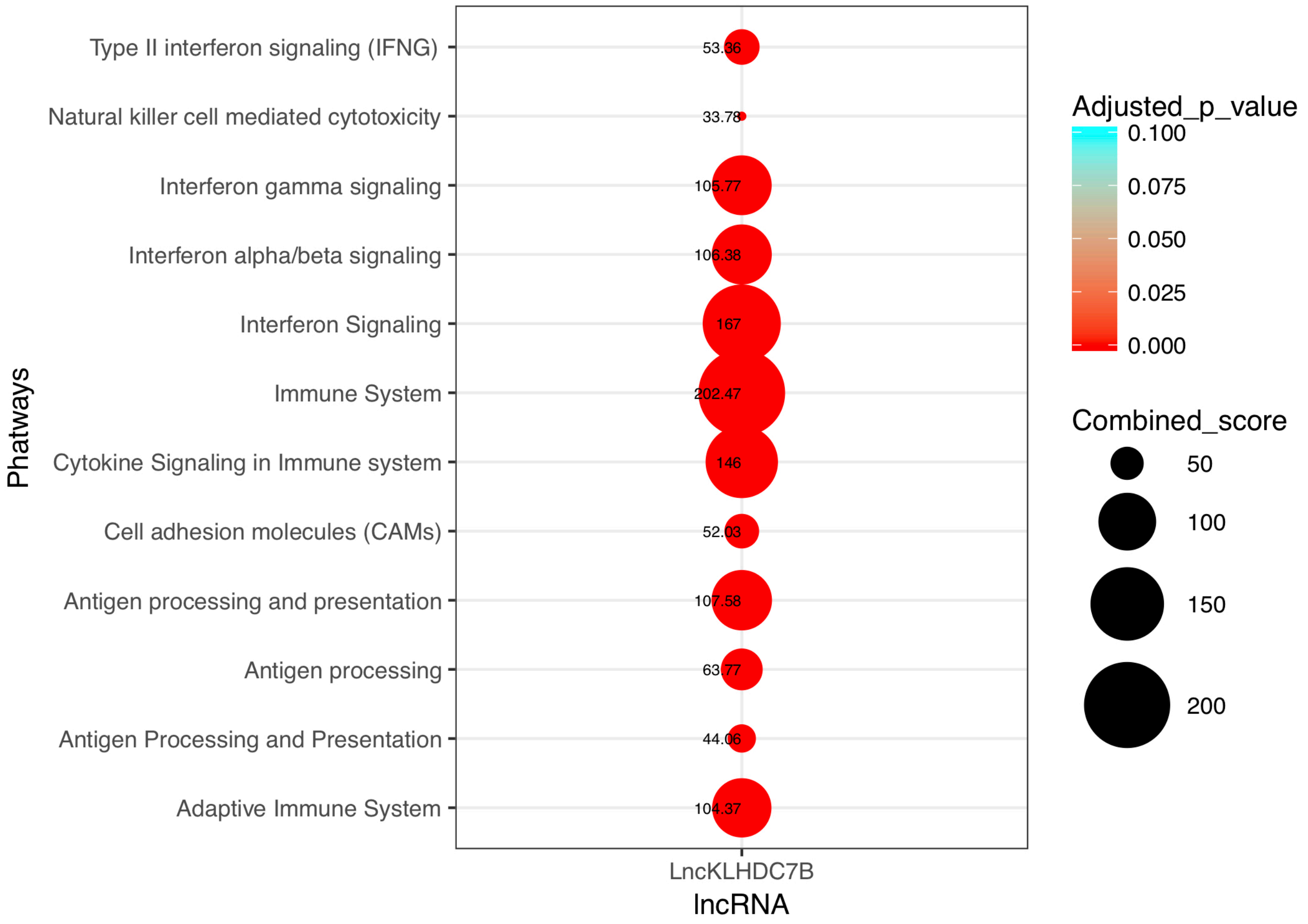

Supplementary figure 5. Guilt-by-association analysis. Bubble chart shows enrichment of biological pathways correlated by expression of LncKLHDC7B. Y-axis label represents pathways. Size and color of the bubble represents the Score each pathway and significance, respectively.

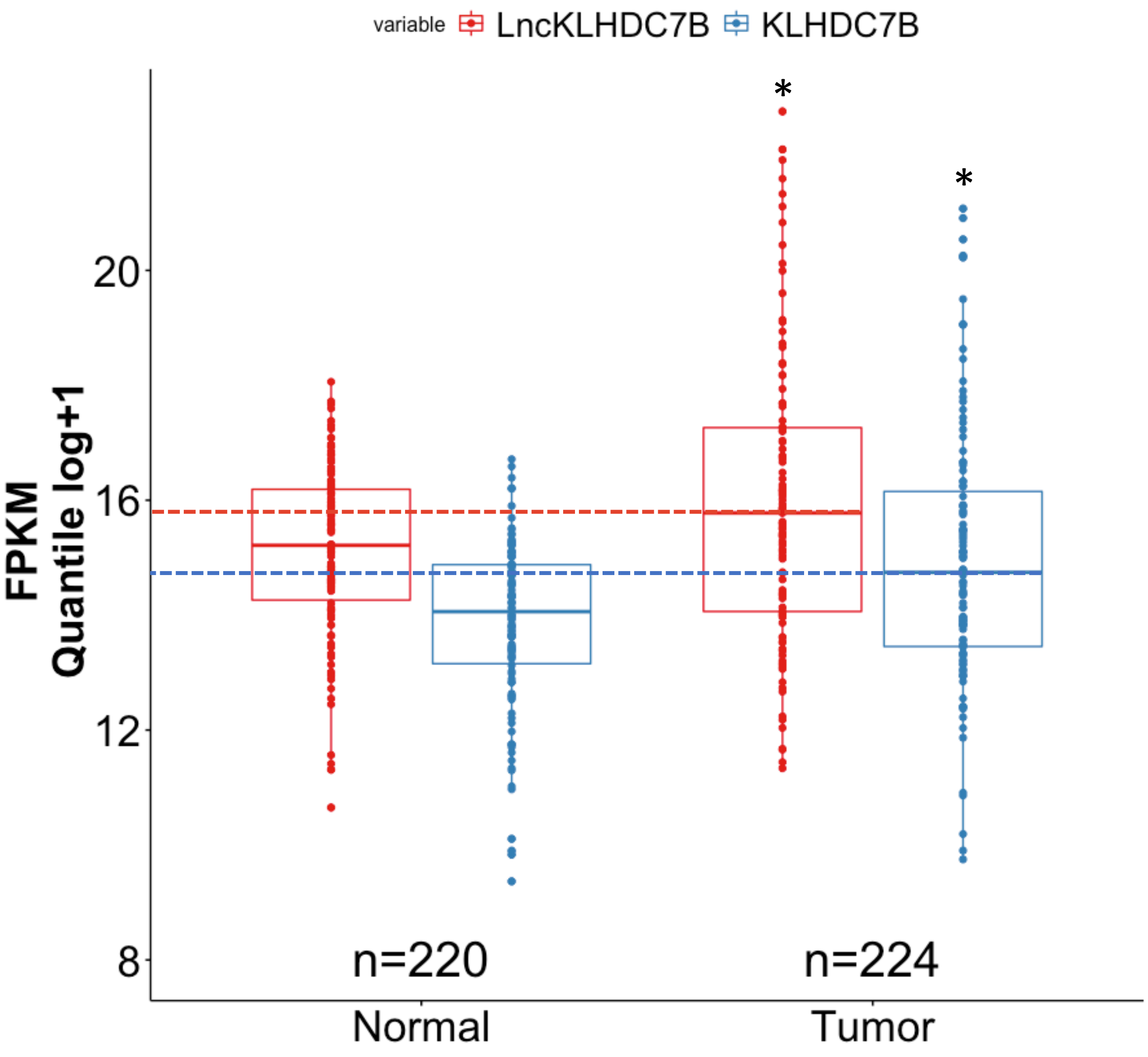

Supplementary figure 6. Up-modulation of LncKLHDC7B and KLHDC7B in tumor samples of breast cancer. Expression of *LncKLHDC7B* and *KLHDC7B* in human normal adjacent and tumor tissues from TCGA data.

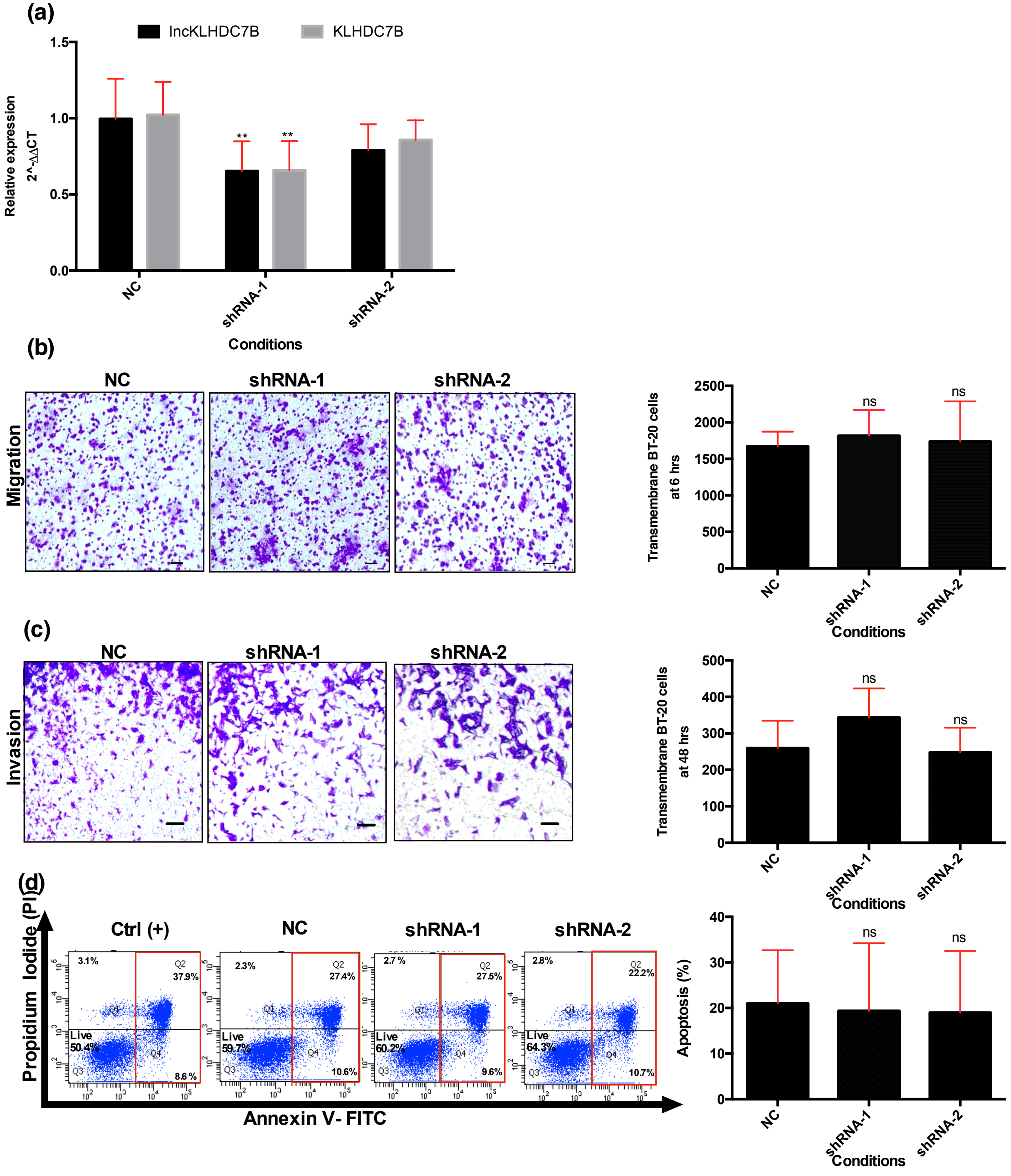

Supplementary figure 7. Functional analysis by the silencing of *LncKLHDC7B* in BT-20 cell line. (a) The expression of *LncKLHDC7B* and *KLHDC7B* in BT-20 cells transfected with NC or shRNA-1 or -2 versus *LncKLHDC7B* was determined by qRT-PCR. (b) Transwell migration and (c) invasion assay showed that *LncKLHDC7B* silencing increase the migration and invasion of BT-20. Representative images are shown on the left and its quantification on the right. Scale bar = 50μm. (d) Flow cytometric analysis of apoptosis (early and late) in BT-20 cell transfected with control and shRNAs-1 and -2 after Annexin-V/PI staining. All data are shown as the mean ± SD of at least three independent experiments. Student's t-test was performed to determine significance, ns = p>0.05, *p<0.05, **p<0.01, ***p<0.001 of NC vs shRNAs.

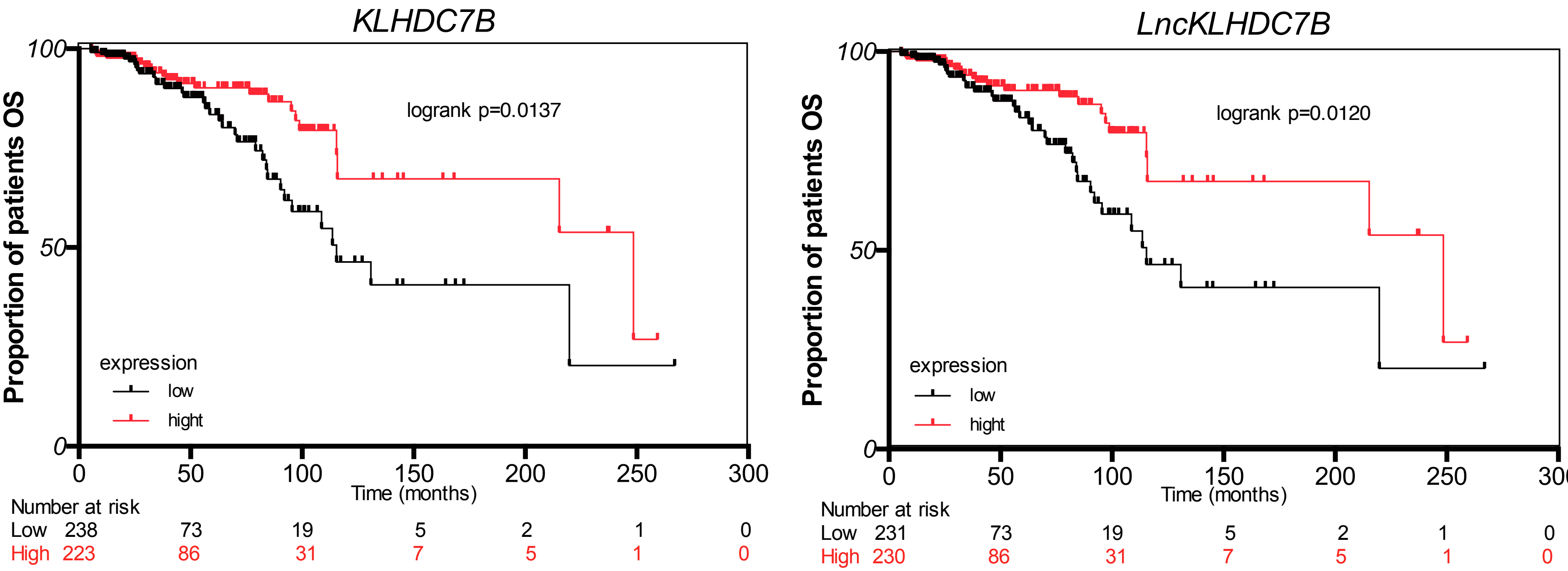

Supplementary figure 8. Kaplan-Meier curve of overall survival (OS). Kaplan-Meier analysis was analyzed according to *KLHDC7B* and *LncKLHDC7B* expression levels from TCGA BRCA data (Anaya, J., 2016. Li et al., 2015). *P* value was obtained using the log-rank test.

References

Cancer Genome Atlas, N., 2012. Comprehensive molecular portraits of human breast tumours. Nature 490, 61-70.

Curtis, C., Shah, S.P., Chin, S.F., Turashvili, G., Rueda, O.M., Dunning, M.J., Speed, D., Lynch, A.G., Samarajiwa, S., Yuan, Y., Graf, S., Ha, G., Haffari, G., Bashashati, A., Russell, R., McKinney, S., Group, M., Langerod, A., Green, A., Provenzano, E., Wishart, G., Pinder, S., Watson, P., Markowetz, F., Murphy, L., Ellis, I., Purushotham, A., Borresen-Dale, A.L., Brenton, J.D., Tavare, S., Caldas, C., Aparicio, S., 2012. The genomic and transcriptomic architecture of 2,000 breast tumours reveals novel subgroups. Nature 486, 346-352.

Lehmann, B.D., Bauer, J.A., Chen, X., Sanders, M.E., Chakravarthy, A.B., Shyr, Y., Pietenpol, J.A., 2011. Identification of human triple-negative breast cancer subtypes and preclinical models for selection of targeted therapies. J Clin Invest 121, 2750-2767.

Liu, Y.R., Jiang, Y.Z., Xu, X.E., Yu, K.D., Jin, X., Hu, X., Zuo, W.J., Hao, S., Wu, J., Liu, G.Y., Di, G.H., Li, D.Q., He, X.H., Hu, W.G., Shao, Z.M., 2016. Comprehensive transcriptome analysis identifies novel molecular subtypes and subtype-specific RNAs of triple-negative breast cancer. Breast Cancer Res 18, 33.

Masuda, H., Baggerly, K.A., Wang, Y., Zhang, Y., Gonzalez-Angulo, A.M., Meric-Bernstam, F., Valero, V., Lehmann, B.D., Pietenpol, J.A., Hortobagyi, G.N., Symmans, W.F., Ueno, N.T., 2013. Differential response to neoadjuvant chemotherapy among 7 triple-negative breast cancer molecular subtypes. Clin Cancer Res 19, 5533-5540.

Anaya, J., 2016. OncoLnc: linking TCGA survival data to mRNAs, miRNAs, and lncRNAs. PeerJ Computer Science 2:e67, 2e:67 ed, PeerJ Computer Science.

Li, J., Han, L., Roebuck, P., Diao, L., Liu, L., Yuan, Y., Weinstein, J.N., Liang, H., 2015. TANRIC: An Interactive Open Platform to Explore the Function of lncRNAs in Cancer. Cancer Res 75, 3728-3737.
